# SMILES-based deep generative scaffold decorator for de-novo drug design

**DOI:** 10.1186/s13321-020-00441-8

**Published:** 2020-05-29

**Authors:** Josep Arús-Pous, Atanas Patronov, Esben Jannik Bjerrum, Christian Tyrchan, Jean-Louis Reymond, Hongming Chen, Ola Engkvist

**Affiliations:** 1 Molecular AI, Hit Discovery, Discovery Sciences, BioPharmaceutical R&D, AstraZeneca, Gothenburg, Sweden; 2 Medicinal Chemistry, Respiratory Inflammation, and Autoimmune (RIA), BioPharmaceutical R&D, AstraZeneca, Gothenburg, Sweden; 3 Department of Chemistry and Biochemistry, University of Bern, Freiestrasse 3, 3012 Bern, Switzerland; 4 Chemistry and Chemical Biology Centre, Guangzhou Regenerative Medicine and Health -Guangdong Laboratory, Guangzhou, China

**Keywords:** Deep learning, Generative models, SMILES, Randomized SMILES, Recurrent neural networks, Fragment-based drug discovery, Data augmentation, RECAP, Matched molecular pairs, Ligand series

## Abstract

Molecular generative models trained with small sets of molecules represented as SMILES strings can generate large regions of the chemical space. Unfortunately, due to the sequential nature of SMILES strings, these models are not able to generate molecules given a scaffold (i.e., partially-built molecules with explicit attachment points). Herein we report a new SMILES-based molecular generative architecture that generates molecules from scaffolds and can be trained from any arbitrary molecular set. This approach is possible thanks to a new molecular set pre-processing algorithm that exhaustively slices all possible combinations of acyclic bonds of every molecule, combinatorically obtaining a large number of scaffolds with their respective decorations. Moreover, it serves as a data augmentation technique and can be readily coupled with randomized SMILES to obtain even better results with small sets. Two examples showcasing the potential of the architecture in medicinal and synthetic chemistry are described: First, models were trained with a training set obtained from a small set of Dopamine Receptor D2 (DRD2) active modulators and were able to meaningfully decorate a wide range of scaffolds and obtain molecular series predicted active on DRD2. Second, a larger set of drug-like molecules from ChEMBL was selectively sliced using synthetic chemistry constraints (RECAP rules). In this case, the resulting scaffolds with decorations were filtered only to allow those that included fragment-like decorations. This filtering process allowed models trained with this dataset to selectively decorate diverse scaffolds with fragments that were generally predicted to be synthesizable and attachable to the scaffold using known synthetic approaches. In both cases, the models were already able to decorate molecules using specific knowledge without the need to add it with other techniques, such as reinforcement learning. We envision that this architecture will become a useful addition to the already existent architectures for de novo molecular generation.
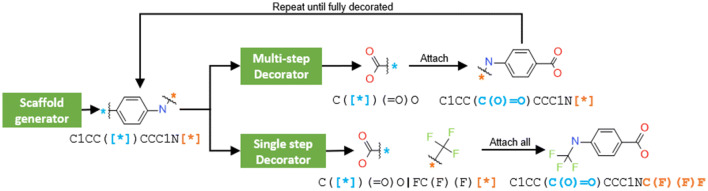

## Introduction

Deep generative models have become a widely used tool to generate new data from limited amounts. They have been applied successfully to generate text [1], images [2], video [3], and music [4]. Additionally, they have been applied to drug discovery and have enabled many new ways to explore the chemical space [5–7]. For instance, Recurrent Neural Networks (RNNs), comprised of several interconnected layers of Long Short-Term Memory (LSTM) cells [8], trained with the ChEMBL database [9] (around 1.8 million compounds), can generate billions of drug-like molecules [10]. Techniques such as transfer learning [11, 12], and reinforcement learning [13] can then be used on the trained model to refine it and obtain molecules of interest (i.e., activity to a particular target, physicochemical property optimization). Other architectures, such as Variational Autoencoders (VAEs) [14, 15], Conditional RNNs (cRNNs) [16], or Generative Adversarial Networks (GANs) [17, 18] have also reported success in generating molecules.

One specific feature of a large number of architectures is the use of the SMILES molecular representation [19]. The use of the full molecular graph has been reported in the literature [20, 21], but SMILES-based models have a simpler architecture and can be trained faster. Moreover, using a randomized SMILES representation [10, 22] instead of a unique (or canonical [23]) during training improves the results substantially and makes the models converge better and overfit less. Nevertheless, up to now, SMILES generative model architectures do not allow molecular generation from scaffolds (i.e., a partially-built molecule with explicit attachment points) mainly due to the restrictions of the SMILES syntax. An approach was published that could generate molecules by completing a SMILES string from both sides using a bidirectional RNN [24], but it is limited to two attachment points. Furthermore, other approaches have been reported that use graph generative neural networks (GGNN), which are even able to decorate a scaffold without the need of specifying attachment points [25, 26]. Unfortunately, GGNNs are experimental architectures that require far more resources to train and sample than SMILES-based architectures [20]. Moreover, a model that is readily integrated to the current SMILES-based generative model pipelines would prove especially useful in a compound series generation [27], where some specific moiety is to be retained, or in library design [28], where synthesizability constraints may apply.

In this article, we describe a deep learning SMILES-based generative architecture that can generate molecules in two steps: first, an RNN that generates scaffolds and then a model (henceforth called a decorator) that generates suitable decorations for each attachment point in the scaffold. A crucial step is to generate training sets that help the model generalize for a wide range of scaffolds. For this reason, an algorithm that exhaustively slices any arbitrary molecular set into a larger set of scaffold-decorations tuples is described. This algorithm bears a resemblance, although it is much more general, to the Hussain-Rea (HR) Matched Molecular Pairs (MMP) [29] algorithm [30] and the one used by Ertl et al. to obtain substituents from molecules [31, 32]. The algorithm can be coupled with randomized SMILES and can generate large amounts of scaffolds and decorations even from small and focused molecular sets, thus serving as a data augmentation technique. Furthermore, the scaffolds and the decorations can be further filtered and only allow those with specific properties. We show that these filterings allow a decorator model to learn specific information on how the scaffolds are to be decorated.

Two experiments were performed to showcase the potential of the model in both medicinal and synthetic chemistry. For the first approach, a set of modulators of the DRD2 receptor obtained from ExCAPE DB [33] were exhaustively sliced using the algorithm described previously. Using an Activity Prediction Model (APM) trained on a larger set of DRD2 active and inactive compounds, we showed that decorator models trained with this dataset were able to obtain predicted active molecular series given a diverse set of scaffolds as input. These series were also plotted on Tree Maps (TMAPs) [34], which cluster molecules according to similarity. The second experiment instead used a subset of drug-like molecules in ChEMBL, which was exhaustively sliced using the same algorithm but restricting the acyclic bonds to cut to those that complied with the synthetic chemistry-based RECAP [35] rules. Results showed that, given a large and diverse set of scaffolds, models trained with the dataset were able to generate decorations that joined to the molecule with a bond fulfilling the RECAP rules. Moreover, the decorations generated were also shown generally to be easily obtainable, making the resulting molecules synthesizable using known routes. An implementation of the architecture alongside the scripts used to slice the training sets are released as open-source software.

## Results

### Architecture overview

**Fig. 1:**
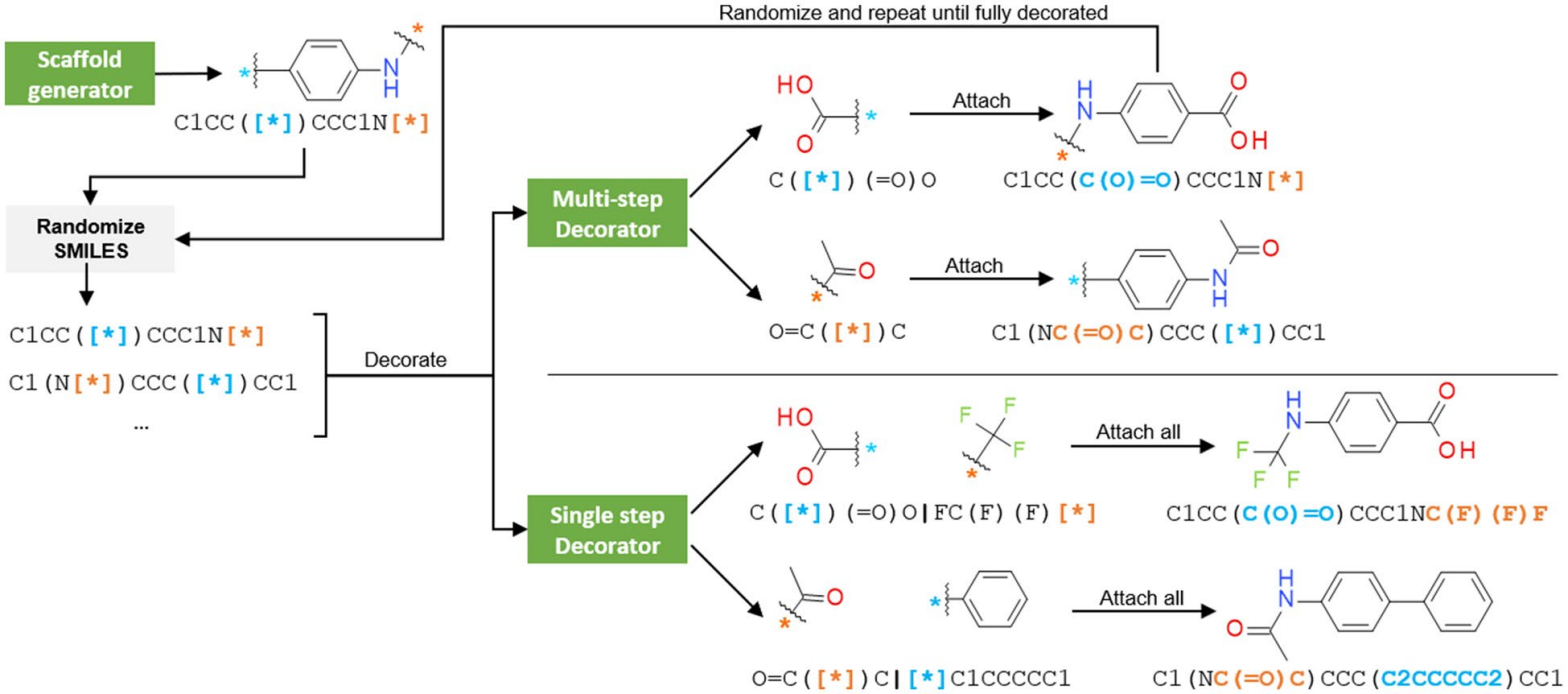
Schema of both the generative model architecture that decorates a scaffold in one step (Single-step decorator) and the one that uses many steps (Multi-step decorator). The first loop is the same for both decorators: a scaffold is created by the scaffold generator, and then some randomized SMILES are obtained. In the single-step decorator, scaffolds are fed to the model, and decorations are generated by order of appearance in the SMILES string. Alternatively, the multi-step decorator generates only the first decoration in the SMILES string, then it is attached to the scaffold, and the process is repeated until all attachment points are decorated

The molecule generation process was divided into two steps: a scaffold generator and a decorator. Both generator and decorator were molecular generative models (see “Methods” for more information) and used the randomized SMILES molecular representation [10]. The SMILES syntax was extended with the token “[*]”, which is supported by some chemistry software libraries, as an attachment point in partially-built molecules. The generation process is summarized in Fig. 1. First, a randomized SMILES of a scaffold was either generated by the scaffold generator or fed in manually. Then, the scaffold was input to the decorator model. Here, two possible decorators were trained, one that decorated only one attachment point at a time and another that decorated all attachment points at once. In the first case, the model decorated the first attachment point in the scaffold SMILES string, the generated decoration was then joined back to the scaffold, and the half-built molecule was fed back in the decorator. The process was repeated until all attachment points were decorated. The randomized SMILES representation of the half-decorated molecules changed at every step, thus moving the relative position of the attachment points in the SMILES string. This process allowed all possible orderings when decorating a molecule with $n$ attachment points to be considered. Alternatively, the model that decorated all attachment points followed a similar process but in only one step, generating all the decorations by order of appearance in the SMILES string using the “|” token as a separator.

### Training set generation

**Fig. 2:**
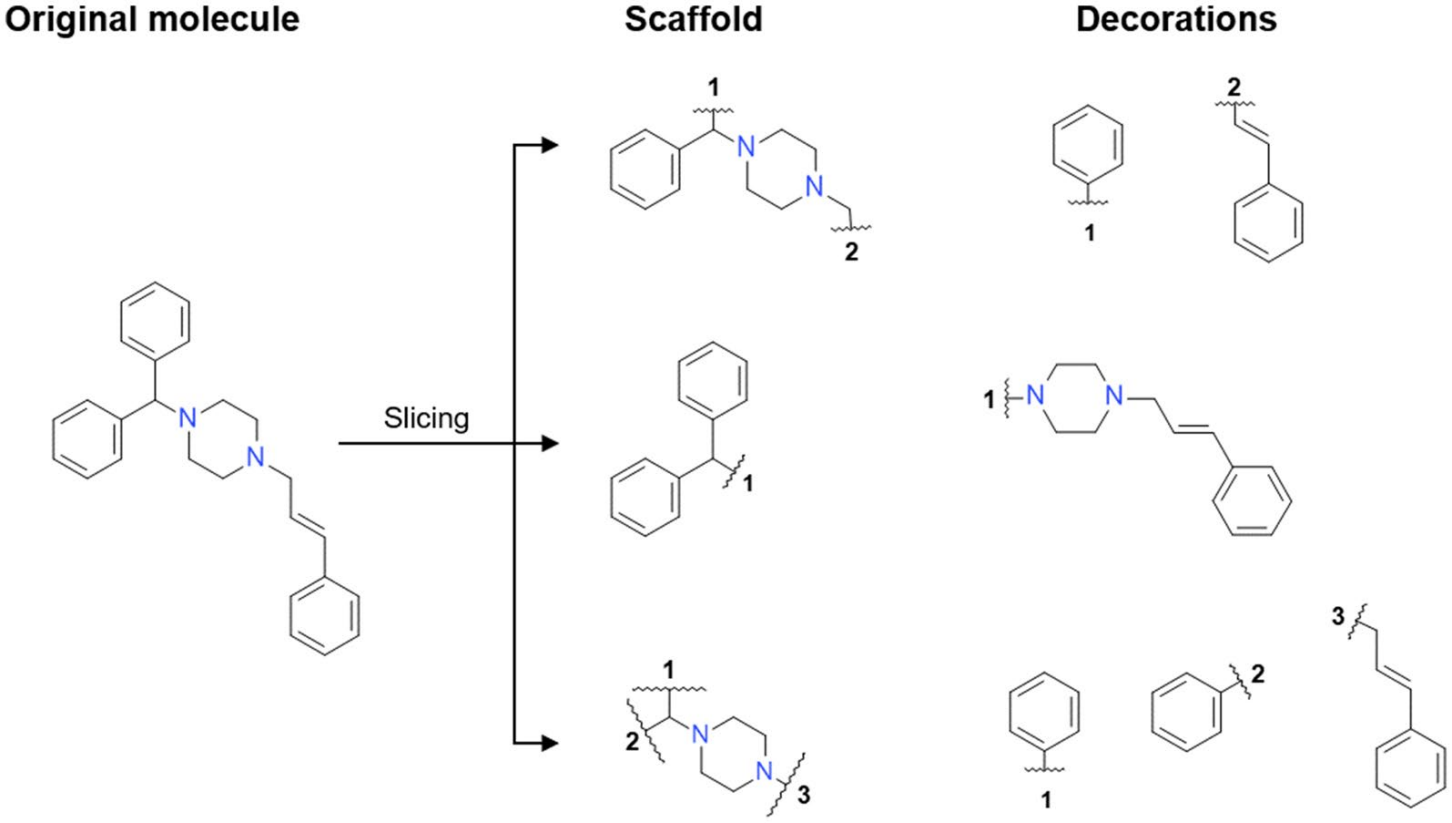
Three possible scaffold-decorations tuples obtained from slicing the DRD2 antagonist *cinnarizine*. The scaffold with the attachment points is on the left and the decorations on the right. Notice that the second case with only one attachment point can also be inverted, converting the decoration to the scaffold and vice versa

A decorator model requires a training set where each item comprised of a scaffold and its decorations. Instead of choosing already created compound series (e.g., patent data), the datasets were created by exhaustively slicing all molecules in a molecular database. For each molecule in the dataset, this algorithm creates fragments by exhaustively removing $c$ single non-cyclic bonds. Then, fragments generated are classified as scaffold and decorations (Fig. 2). From a slicing of a molecule, if there is no fragment with $attachments = c$, the combination is discarded. Given that each molecule can be sliced in many different ways, this approach generally yields more than one scaffold per molecule. Additionally, a scaffold can have more than one set of decorations, each of them from a different molecule. Each of these combinations, henceforth called scaffold-decorations tuple, is comprised of a scaffold and as many decorations as attachment points the scaffold has.

By default, the algorithm has all acyclic bonds as candidates for slicing, but those can be further filtered. Additionally, the scaffolds and decorations can be filtered whether they match specific physicochemical properties. Specifically, in all training sets used in this research, the scaffolds had at least one ring, and the decorations had to comply with the rule of 3 [36] (i.e., $MolWeight \le 300 Da; HBD \le 3; HBA \le 3;ClogP \le 3;RotBonds \le 3$).

### Generating predicted active molecular series on DRD2

The primary purpose of a scaffold decorator is to decorate many times, as meaningfully as possible, any input scaffold. In the first experiment, a small dataset, comprised of 4211 Dopamine Receptor D2 (DRD2) active modulators, was used to train decorator models (both multi-step and single-step). Then, these models were tested on a diverse set of scaffolds, both similar and different from the training set data. From each scaffold, a molecular series was obtained, which was then compared to randomly decorated molecules using an activity prediction model (APM).

#### Preparation of the dataset

**Fig. 3:**
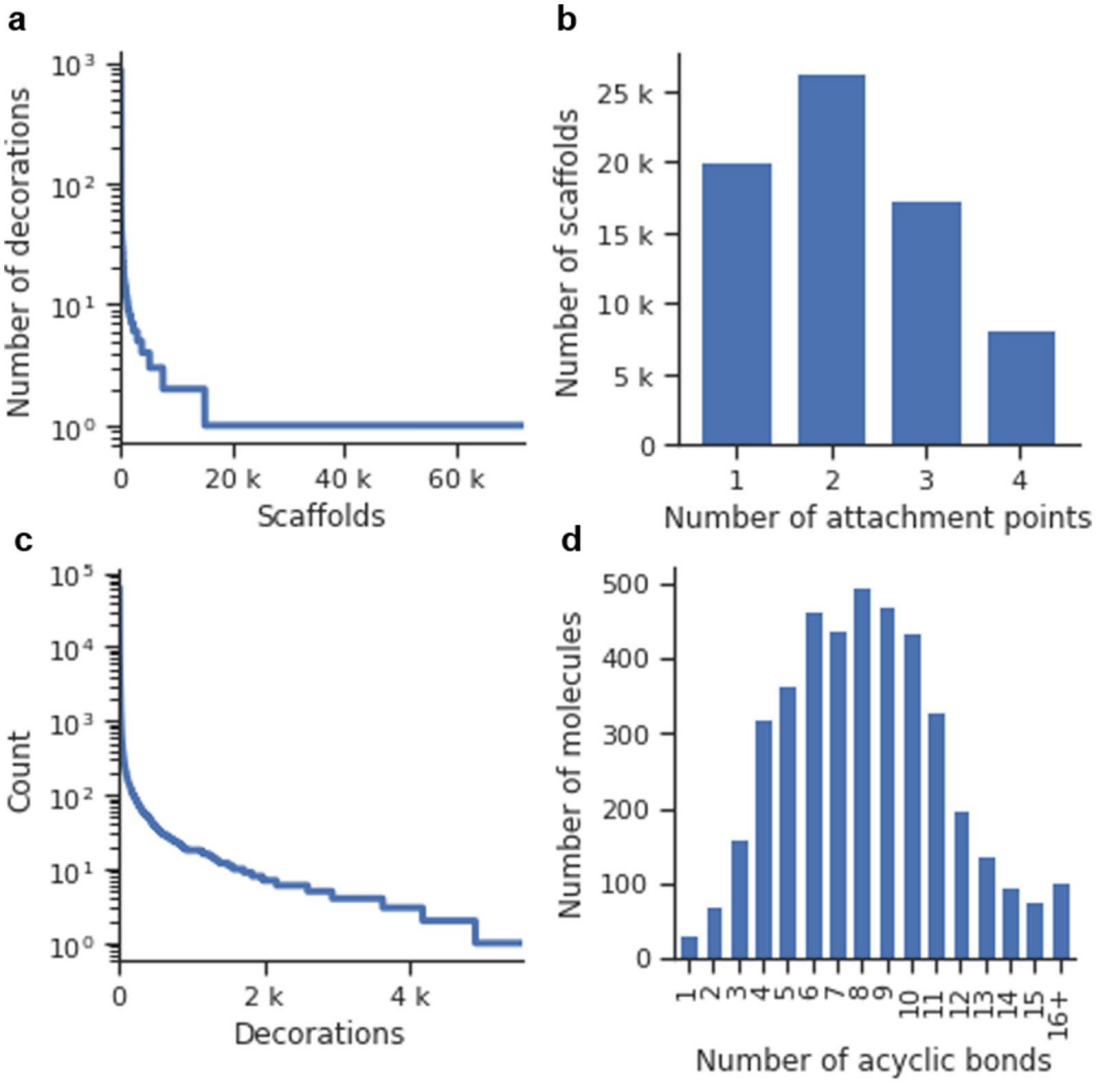
Plots describing the resulting set of 137,061 scaffold-decorations tuples obtained from slicing a set of 4211 DRD2 modulators (see “Methods”). **a** The number of decorations per scaffold in the dataset ordered from right to left (notice that the y-axis is in logarithmic scale). **b** Histogram of the number of attachment points of the entire set. **c** The number of times each decoration appears in the dataset ordered from right to left (notice that the y-axis is in logarithmic scale). **d** Histogram of the number of acyclic bonds per molecule

A dataset comprised of 4211 Dopamine Receptor D2 (DRD2) active modulators ($pXC_{50} \ge 5$) obtained from ExCAPE-DB [33] (Additional file 1: Methods S1) was processed the following way: First, all molecules were exhaustively sliced using the algorithm mentioned in the previous section. This processing step yielded a total of 137,061 scaffold-decorations tuples, which included 72,010 unique scaffolds with up to four attachment points. The distribution of the number of decorations per scaffold showed an exponential nature (Fig. 3a), and most (56,933–79.1%) of the scaffolds were singletons (i.e., only one set of decorations). Also, the number of attachment points per scaffold was plotted and showed that most scaffolds had two attachment points (Fig. 3b). The training set was comprised of 5532 unique decorations, also distributed exponentially (Fig. 3c). Both a scaffold generator model and decorator models were trained (Additional file 1: Methods S2 for more details on the training process).

#### Analysis of validation set scaffolds

**Fig. 4:**
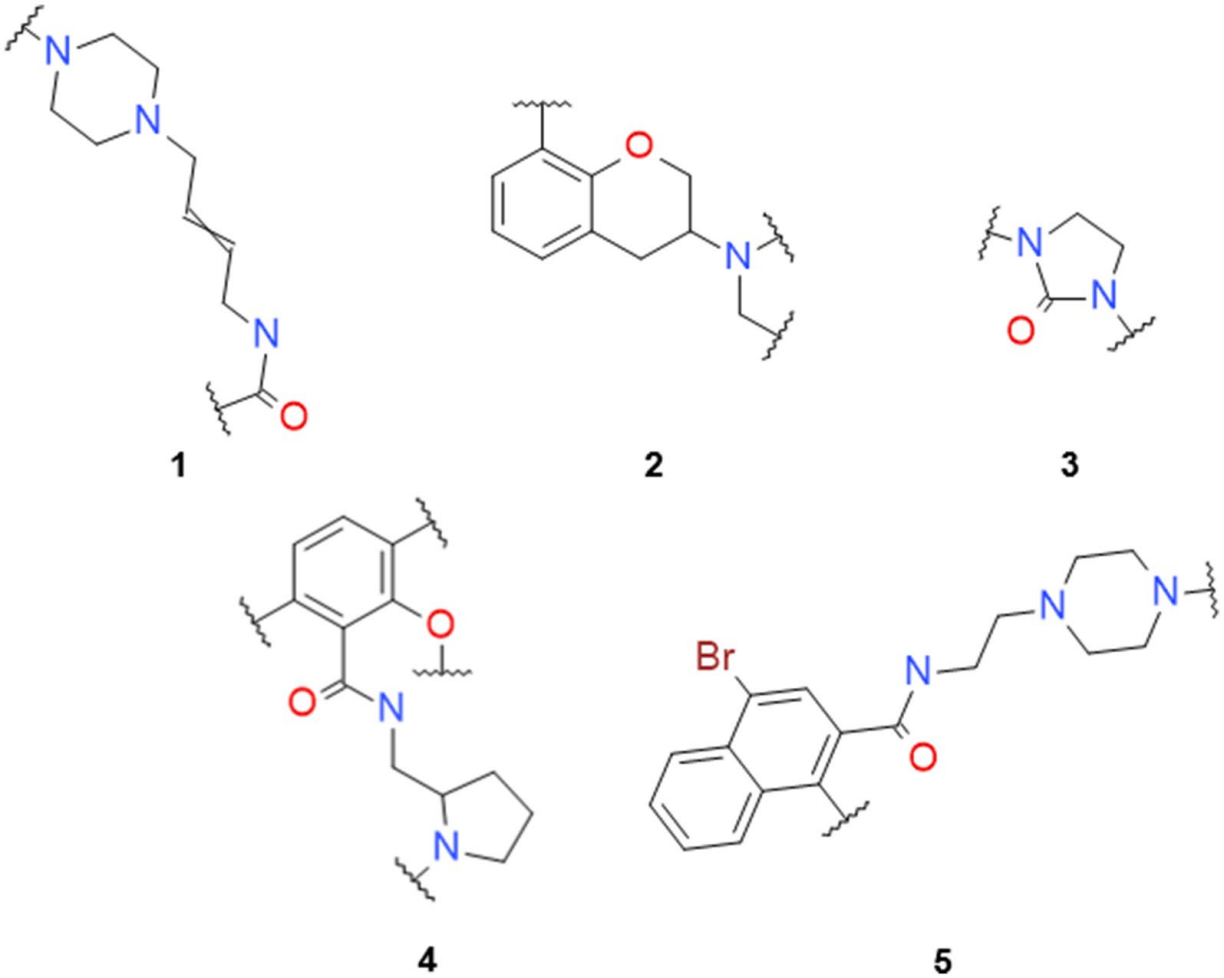
Five scaffolds only present in the validation set of the scaffold decorator model

A validation set comprised of 5532 scaffold-decoration tuples was extracted by removing all tuples with five randomly selected scaffolds (Fig. 4) alongside all tuples obtained from any of the 152 molecules with those scaffolds. The five selected scaffolds were then decorated multiple times with the multi-step decorator model, yielding a total of 14,300 unique molecules, which included 63 (41.4%) molecules present in the validation set (Table 1). Moreover, the model was able to decorate the scaffolds in many more ways and generate thousands of molecules. Most of these were predicted active by an activity prediction model (APM), which was trained from a larger set of DRD2 active and inactive molecules (see “Methods”). Next, randomly generated decoys were sampled for each scaffold. These decoys were obtained from decorating each scaffold with drug-like fragments from ChEMBL, maintaining the same molecular weight distribution as the training set. For each scaffold, as many decorators were generated as times the scaffold decorator model was sampled (Additional file 2: Table S2 for the exact numbers). Results showed that the percent of predicted active molecules was always lower than that obtained from the decorated molecules (Table 1, left). Furthermore, the same decoy generation procedure was done this time using decorations from the training set and, even though there was a higher percentage of predicted active molecules compared to the ChEMBL decoys, it was lower than the one from the set obtained with the decorator model (Table 1, right). A comparison of the generated decoys and decorated molecules was also carried out and showed that the overlap between the decoy sets and the generated molecules was minimal (Additional file 2: Table S2). Specifically, the ChEMBL decoys had no overlap whatsoever in scaffolds (3), (4), and (5) and just nine molecules in total for the other two scaffolds. The overlaps with the DRD2 decoys were slightly higher, but only 31 molecules (0.2% of all generated molecules) among all scaffolds. Lastly, there was a substantial difference in the number of decorations sampled with each scaffold: in scaffolds (4) and (5), less than a thousand different molecules were obtained, whereas in (1), (2) and especially (3) many more molecules were generated.

**Table 1:** Results from the decoration process of the DRD2 molecules on the five validation set scaffolds

S	Generated		ChEMBL decoys			DRD2 decoys		
	Total	% act.	% act.	% diff	EOR	% act.	% diff	EOR
1	1728	64.5	0.9	63.6	67.7	4.2	60.3	15.5
2	1626	52.4	8.3	44.1	6.3	19.7	32.7	2.7
3	10,666	35.6	0.8	34.8	46.3	16.2	19.4	2.2
4	262	84.7	0.4	84.3	236.1	0.9	83.8	89.0
5	18	88.9	7.8	81.1	11.3	10.1	78.8	8.8

Total number of molecules sampled (Total); percent of generated molecules that are predicted as active ($p_{active} \ge 0.5$) by the APM (% act); for both the decoys decorated with ChEMBL fragments and DRD2 fragments from the training set: percent of predicted active decoys ($p_{active} \ge 0.5$) (% act); difference between the generated predicted active percent and the predicted active percent of the decoys (% Diff); Enrichment Over Random ($percent_{active} /percent_{decoy}$) (EOR)

#### Generating molecules from new scaffolds

**Fig. 5:**
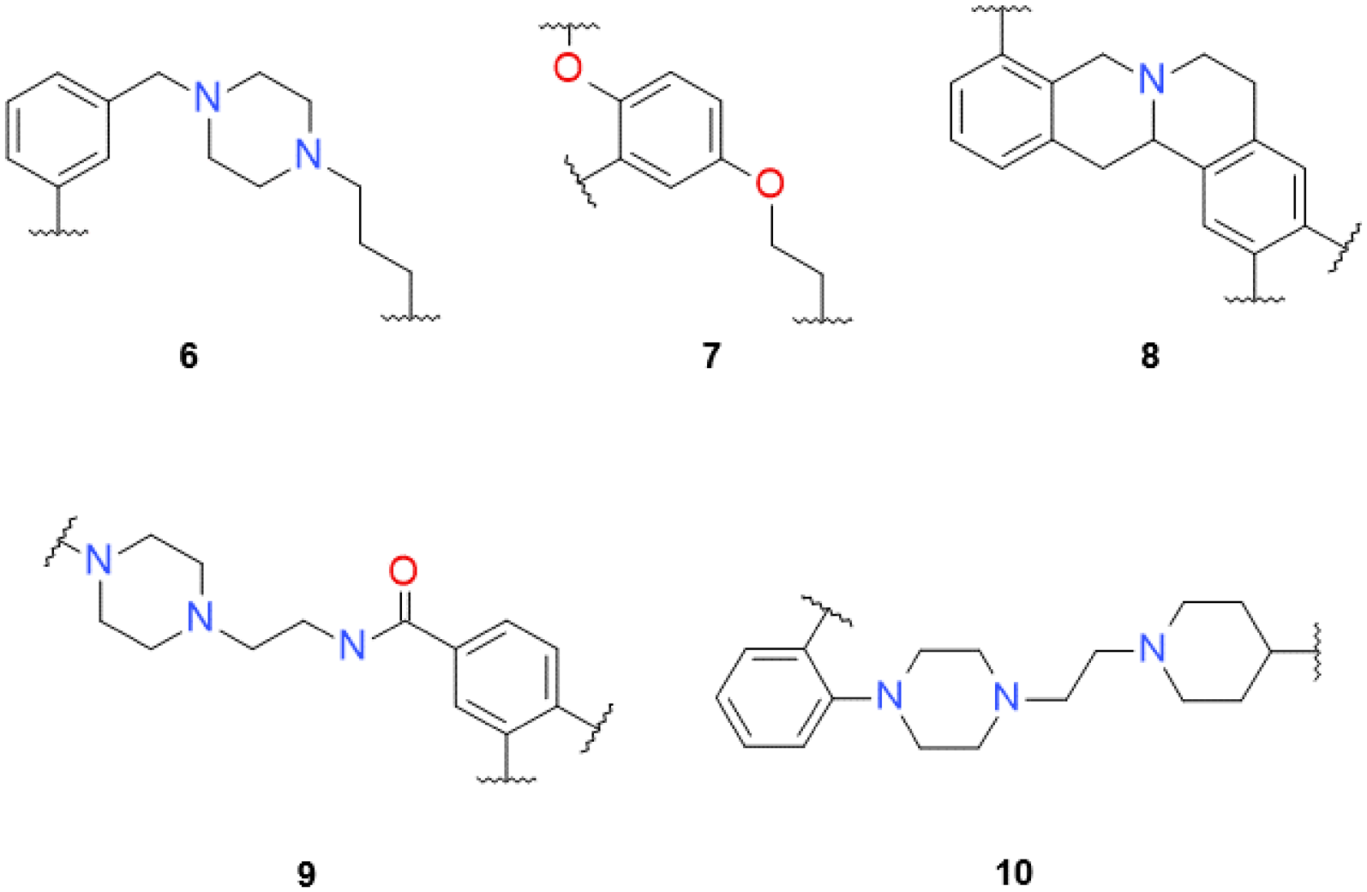
Five scaffolds generated from a scaffold generative model (non-dataset scaffolds, see “Methods” for more information)

The complete generative process, as shown in Fig. 1, was performed by first obtaining five diverse scaffolds not present in the DRD2 dataset (non-dataset scaffolds) (Fig. 5). They were sampled from a molecular generative model trained with the scaffolds from the training set (see “Methods”) and were then decorated multiple times using the multi-step scaffold decorator model. Results showed that the ratio of generated molecules predicted as active is generally very high, going from 45.4 to 98.9% (Table 2). But most importantly, the decorator molecules always had a higher ratio of predicted active molecules than both the ChEMBL and training set decoy sets. Notice that the more complete a scaffold is, the higher the number of predicted active decoys appear. This particularity is noticeable in scaffolds (6), (8), and especially (10) and may point to applicability domain limitations of the APM. Moreover, the absolute number of different molecules decorated varied greatly among scaffolds, and the total number of molecules (26,140) was approximately two times greater than that of the previous section, possibly because the model had less information from the scaffolds and was less focused. Lastly, the overlap between the decoys and the generated molecules was also calculated and yielded higher results to those in the previous section (Additional file 2: Table S3). A total of 167 molecules (0.6% of all generated molecules) were also ChEMBL decoys, and 411 molecules (1.6% of all generated molecules) were also training set decoys.

**Table 2:** Results of the decoration for each of the non-dataset scaffolds

S	Generated		ChEMBL decoys			DRD2 decoys		
	Total	% act.	% act.	% diff	EOR	% act.	% diff	EOR
6	1864	78.3	49.5	28.9	1.6	66.6	11.7	1.2
7	15,724	45.4	1.0	44.4	44.2	10.6	34.8	4.3
8	2178	80.2	44.3	35.9	1.8	49.3	30.9	1.6
9	5362	85.4	3.1	82.3	27.9	7.0	78.4	12.2
10	1012	98.9	90.4	8.4	1.1	93.7	5.2	1.1

Total number of molecules sampled (Total); percent of generated molecules that are predicted as active ($p_{active} \ge 0.5$) by the APM (% act); For both the decoys decorated with ChEMBL fragments and DRD2 fragments from the training set: Percent of predicted active decoys ($p_{active} \ge 0.5$) (% act); difference between the generated predicted active percent and the predicted active percent of the decoys (% Diff); Enrichment Over Random ($percent_{active} /percent_{decoy}$) (EOR)

#### Decorating scaffolds in one step

**Fig. 6:**
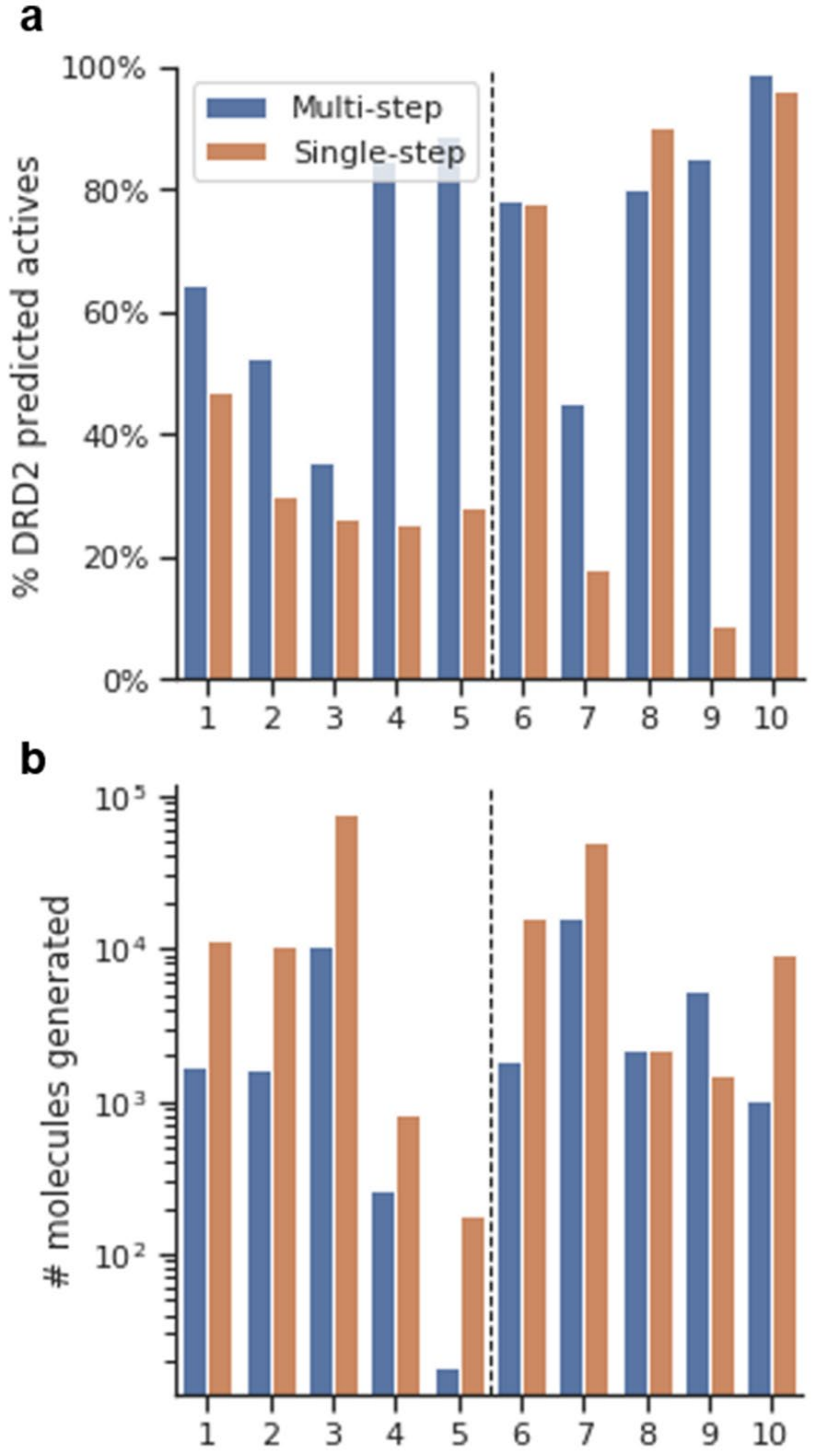
Bar plots comparing the multi-step (blue) and single-step (orange) decorator models for all 10 scaffolds (left, validation set; right, non-dataset. **a** Plot of the  % DRD2 predicted actives. **b** Plot of the number of generated molecules. Notice that the y-axis is logarithmic

A decorator model that decorated all attachment points in a single step was also trained with the same hyperparameter configuration, training, and validation sets as the previous one. Results showed that the single-step architecture model was able to generate 90 out of 152 molecules (59.2%) from the validation set. This result, although better than the multi-step model, must be understood with care. A comparison between the multi-step and single-step models must be made in relative terms, as the nature of the multi-step architecture sampling process does not allow controlling how many molecules are going to be sampled. On the other hand, the decorator showed an overall lower percent of predicted active molecules in the APM benchmark for all validation set scaffolds (Fig. 6, Additional file 2: Table S1 for all data). When being decorated with the non-dataset scaffolds (6) to (10), the results showed a similar trend (Additional file 2: Figure S4 for all data). The number of molecules obtained was more than eight times higher when decorating scaffolds (1) to (5) (100,685 molecules) and around three times higher when decorating the non-dataset scaffolds (6)–(10) (78,681 molecules). These results were highly influenced by the sampling procedure used in both models, but also indicated that the single-step model was less focused and was able to generate more diversity.

#### Analysis of the generated molecular diversity

The diversity of the decorated molecules for each scaffold in both decorator architectures was analyzed in two ways. Firstly, four plots of the molecular weight, cLogP, Synthetic Accessibility (SA) Score, and Quantitative Estimate of Drug-likeness (QED) were calculated on even samples of molecules from the training set, generated molecules from both scaffold sets and both decoy sets (Additional file 2: Figures S1 and S2). These plots showed that the molecules generated with decorator models tended to follow more the training set distribution than the decoys. This effect was especially noticeable on the cLogP and the SA Score, and slightly less in the QED plot. The molecular weight distributions of all subsets were similar, except that of the non-dataset scaffolds of the single-step decorator architecture.

**Fig. 7:**
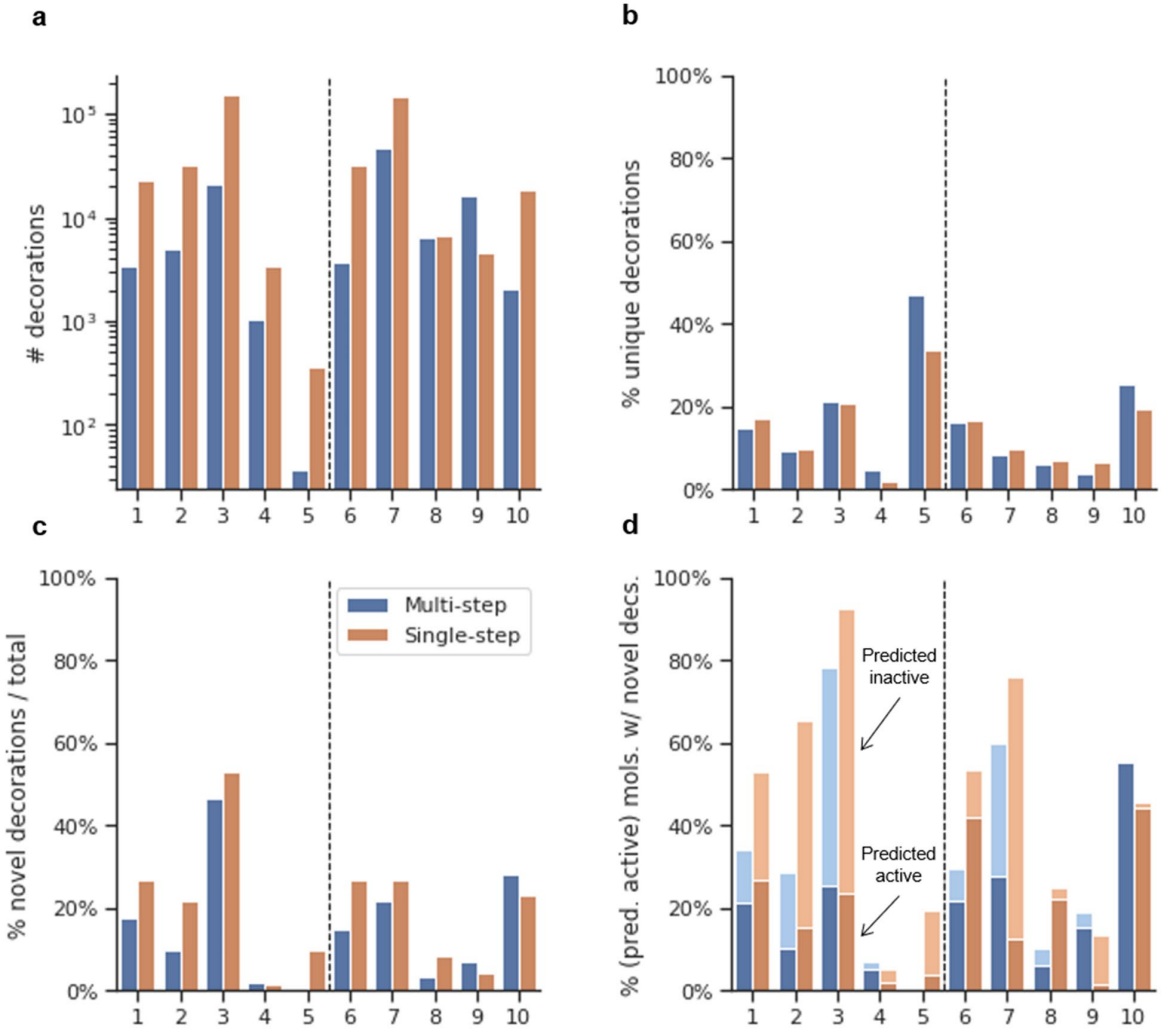
Four bar plots describing properties for molecules generated from scaffolds (1) to (10) using both the multi-step (blue) and single-step (orange) decorator models. **a** The absolute number of decorations (including repeats) generated (notice that the y-axis is in logarithmic scale); **b** percentage of unique decorations; **c** percentage of decorations not present in the training set (in absolute numbers); **d** percentage of generated molecules that have at least one novel decoration. In darker color, the subset thereof that is predicted as active using the DRD2 APM

Secondly, the decorations generated with the decorator models were compared for novelty (Fig. 7). Results showed that scaffolds differed significantly on the number of decorations generated (Fig. 7a). For instance, molecules generated from scaffolds (3), (7), and especially (9) have more than an order of magnitude more decorations than the rest. Alternatively, scaffolds (4) and especially (5) have a tiny number. This difference is not due to the number of attachment points, as (4) has four, and (3) has only two. Additionally, the molecules obtained from the single-step model have more abundance of different decorations and can be attributed to the larger sampled size. Interestingly, when checking the percentage of unique decorations, there is no substantial difference between the multi-step and single-step models (Fig. 7b). Novelty is analyzed in Fig. 7c, d and shows that, albeit only ~ 20% of the decorations of molecules generated are novel on average, a much larger percentage of the molecules include at least one novel decoration. This finding indicates that novel decorations are added to only some attachment points on each scaffold, probably in positions where there is more training information from which to generalize. Lastly, from molecules with at least one novel decoration, a large number of them are predicted as active by the model, meaning that the decorations added tend to do not negatively affect the activity prediction of the molecule.

#### TMAP visualization of molecular series

**Fig. 8:**
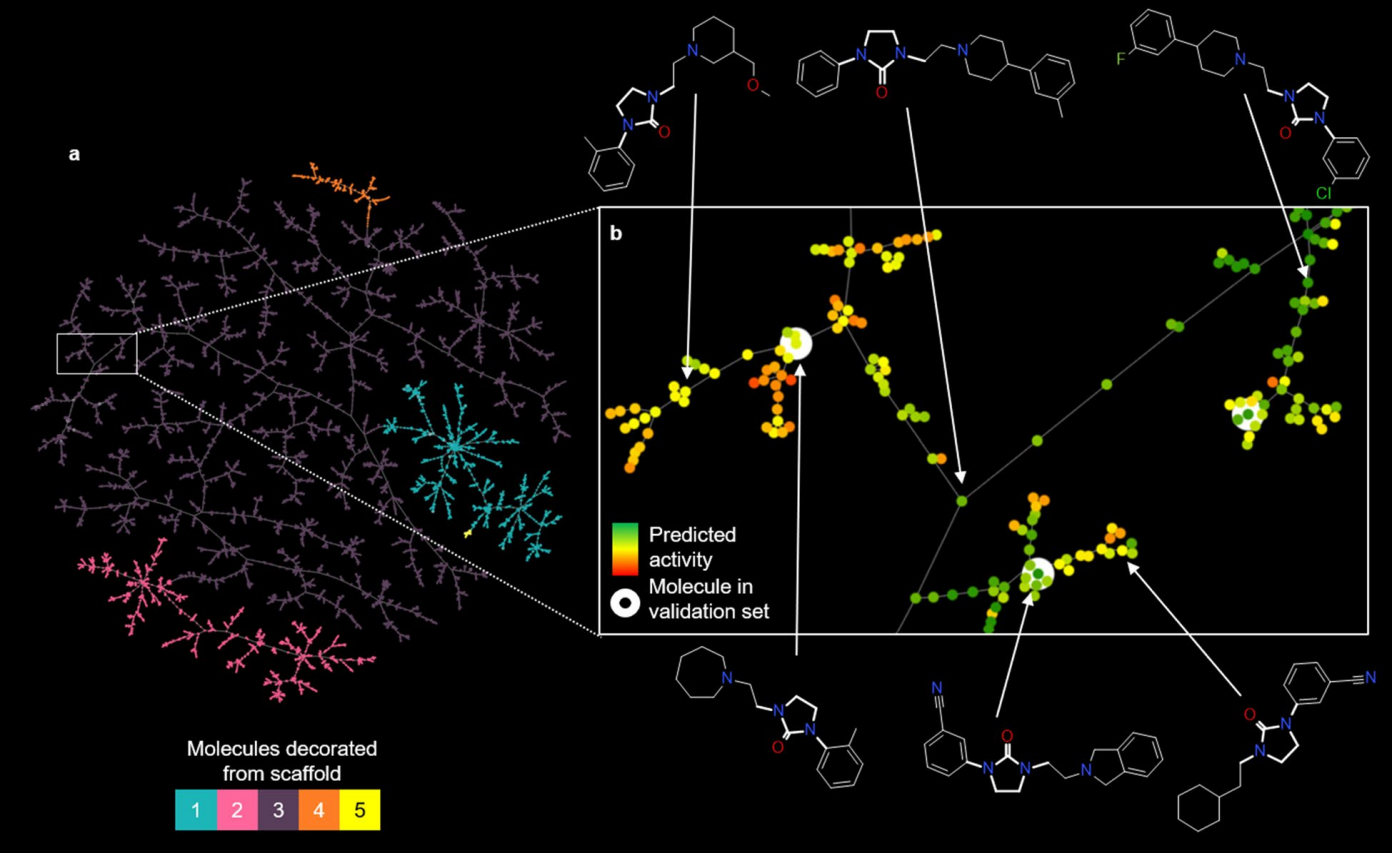
TMAP of the 14,300 molecules obtained by decorating scaffolds (1) to (5) using a multi-step decorator model. **a** Overview of the whole TMAP colored by scaffold. **b** A zoomed-in version of a small section colored by predicted activity on DRD2 (red–yellow–green) and highlighting in white the molecules present in the validation set. Some molecules, pointed to their position in the TMAP with arrows, have been drawn as an example

Tree Maps (TMAPs) [34] are a technique for unsupervised visualization of high dimensional data that creates a 2D layout of a minimum spanning tree constructed in the original space. We used this tool to visualize structural similarity among generated molecules. Each map shows the compounds as dots with up to three concentric circles: the first depicts activity (0–1; from red through yellow and to green), the second depicts the scaffold, and the optional third circle depicts whether the compound is found in the dataset. This last circle only appears in the models that use scaffolds from the validation set. The TMAP generated for the molecules obtained from scaffolds (1) to (5) is shown in Fig. 8. First, notice how the TMAP generally clusters without supervision the molecules from each scaffold (Fig. 8a). Next, when the TMAP is zoomed in (Fig. 8b), each of the terminal branches of the tree represents molecules (colored by predicted activity) with close similarity values. These are generally similar to the validation set molecules (highlighted in white), but sometimes more diverse decorations are generated. Interactive TMAPs of both generated molecules and decoys for scaffolds (1) to (5) and (6) to (10) for both the multi-step and the single-step decorator models are available online.

### Decorating scaffolds with a synthetic chemistry-aware model

In the second experiment, instead of training a model with a small training set, the ChEMBL database [9] was employed and filtered in a way that a decorator model was only trained with drug-like scaffolds and decorations joined only by bonds that comply with the synthetic chemistry RECAP rules [35]. This change forced the model to work following those specifications and to decorate each scaffold with decorations that were both drug-like and attachable to the scaffold using known synthetic routes.

#### Preparation of the dataset

**Fig. 9:**
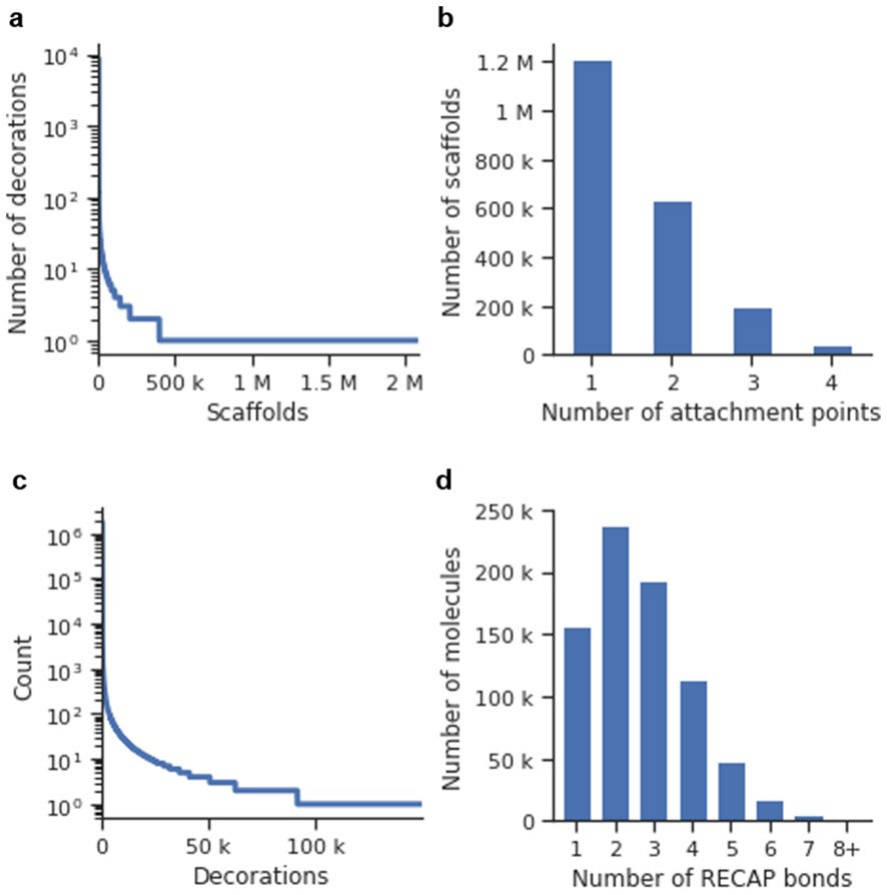
Plots describing the resulting 4,167,207 scaffold-decorations tuples obtained from slicing 827,098 drug-like molecules from ChEMBL (see “Methods”). **a** The number of decorations per scaffold in the dataset ordered by the scaffolds that have the most decorations to the scaffold that has the least (notice that the y-axis is in logarithmic scale). **b** Histogram of the number of attachment points of the entire set. **c** The number of times each decoration appears in the dataset ordered from left to right (notice that the y-axis is in logarithmic scale); **d** Histogram of the number of RECAP bonds per molecule in the training set

The ChEMBL 25 database was filtered down (Additional file 1: Methods S1) to a drug-like set of 827,098 molecules, and the same slicing algorithm used before was applied with the additional restriction that only bonds that complied with the RECAP rules were candidates to be sliced. Moreover, scaffold-decorations tuples were filtered to only allow those that all decorations were fragment-like. This slicing yielded a total of 4,167,207 scaffold-decorations tuples, which included 2,080,212 unique scaffolds. Even more than in the DRD2 set, most scaffolds were singletons (1,682,891–80.9%), and their abundance followed a power-law distribution (Fig. 9a).

The resulting set was comprised mostly of scaffolds with only one attachment point on average (Fig. 9b). This situation was caused by the data augmentation factor being much lower than in the DRD2 set, as there were only two RECAP bonds per molecule on average (Fig. 9c) compared to the nine acyclic bonds per molecule in the DRD2 set (Fig. 3c). Lastly, the set contained 149,530 unique decorations, and their occurrence distribution was also exponential (Fig. 9d). Both a scaffold generator and decorator models were trained (Additional file 1: Methods S2 for more details on the training process).

#### Decorating validation set and non-dataset scaffolds

As in the previous experiment, two sets of scaffolds were collected: a set of 42 scaffolds only present in the validation set (amounting to 5295 molecules not present in the training set), and a set of 40 scaffolds obtained from a generative model not present in the ChEMBL dataset (non-dataset scaffolds—see “Methods” for more information). Both sets of scaffolds were decorated multiple times with the multi-step decorator model, yielding a total of 12,294 and 11,504 different molecules per scaffold on average, respectively.

**Fig. 10:**
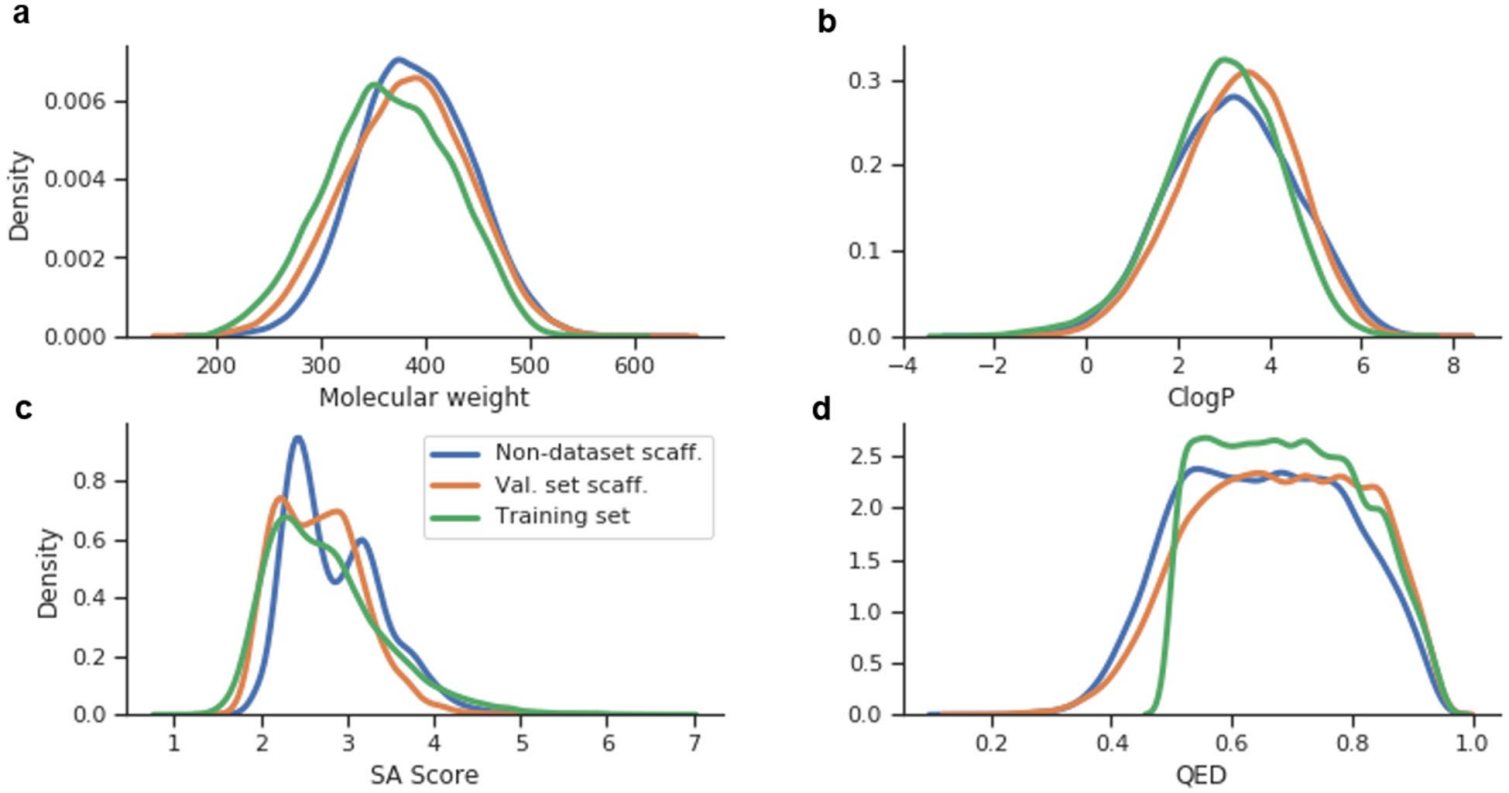
Histograms of different descriptors calculated in three sets of molecules obtained from the ChEMBL multi-step decorator model: Generated molecules from non-dataset scaffolds (blue), generated molecules from validation set scaffolds (orange) and training set molecules (green). **a** Molecular weight (Da); **b** ClogP; **c** Synthetic Accessibility Score; **d** Quantitative Estimate of Drug Likeness (QED). Notice that one of the filtering conditions of the ChEMBL subset was that the molecules had $QED > 0.5$

The decorated molecules from the validation set scaffolds included 35.4% of the validation set decorations, a result slightly lower than in the DRD2 experiment. The quality of the generated molecules was checked by plotting several descriptor distributions and comparing them to the training set distribution. Results show that all descriptor distributions (Fig. 10) were similar and indicated that the decorator model was able to create molecules that, apart from fulfilling the RECAP rules in the attachment points, were equivalently drug-like and synthesizable given any scaffold.

Different properties related to synthesizability were calculated for the molecules generated from both validation set and non-dataset scaffolds (Table 3top). For the molecules generated from validation set scaffolds, results showed that nearly 98% of molecules had all bonds joining the scaffolds with the decoration following RECAP rules. Additionally, 82.7% of the molecules had all decorations included in the training set, and, when allowing for one decoration to be different than training set decorations, the percentage increased to 99.6%.

**Table 3:** Descriptors calculated for the molecules decorated with both model architectures (single and multi-step) from the validation set scaffolds and the non-dataset scaffolds

Set	Mols/scaff.	A (%)	B (%)	C (%)	D (%)	E (%)
*Multi-step decorator model*						
Validation set scaff.	12,294	97.9	82.7	99.6	68.3	98.2
Non-dataset scaff.	11,504	99.2	89.4	99.8	78.8	98.7
*Single-step decorator model*						
Validation set scaff.	38,344	95.9	63.2	99.6	52.7	98.5
Non-dataset scaff.	25,462	97.9	66.1	99.8	57.3	98.7

Molecules per scaffold (Mols/scaff.); See the list above for information on the other fields

*A* Percent of decorated scaffolds with all attachment point bonds RECAP compliant

*B* Percent of decorated scaffolds with all decorations in the training set

*C* Percent of decorated scaffolds with at most one decoration not in the training set

*D* Percent of decorated scaffolds with all decorations in ZINC in-stock

*E* Percent of decorated scaffolds with at most one decoration not in ZINC in-stock

The decorator model mostly decorated with already seen decorations but sometimes created new ones. Furthermore, the same percentages were calculated against the ZINC [38] fragment-sized in-stock subset molecular database (Additional file 1: Methods S1) and reached 98.2% of molecules with at most one decoration not present in ZINC. Very similar results were obtained for the set of molecules generated from non-dataset scaffolds.

#### Decorating scaffolds in one step

A single-step decorator model was also trained using the same hyperparameters and training set as the multi-step decorator. The same sets of scaffolds were decorated, and when the values described in the previous section were calculated showed that performance was slightly worse overall (Table 3, bottom). The decorated molecules from the validation set scaffolds included 55.2% of all molecules in the validation set. This result was much higher, but it could be attributed to the sampling method used in the single-step architecture. On the other hand, the percent of molecules with valid RECAP bonds joining the scaffold with the decoration decreased by 2–4% compared to the multi-step model. Moreover, the properties of the generated molecules did not follow the training set properties as well as those generated with the multi-step model (Additional file 2: Figure S4). In contrast, the ratio of novelty was ~ 20% higher: 30–35% of molecules generated contained at least one decoration not present in the training set, and 40–45% of molecules had at least one decoration not present in ZINC. This result showed that, as in the DRD2 experiment, the single-step architecture was much less focused. Lastly, TMAPs of a sample of all the molecules from five randomly selected scaffolds were generated for both models and scaffold sets and are available at the Reymond group website.

## Discussion

### Context-sensitive decoration of scaffolds allows for controlled chemical space exploration

Traditional computational drug discovery approaches obtain novel candidates by generating large molecular libraries, which are then filtered and characterized using computational means (e.g., QSAR [37], docking [38]). Another common approach uses metaheuristics (e.g., genetic algorithms) to explore the chemical space and intelligently find novel active compounds [39]. Both approaches are based on creating large amounts of molecules on-the-fly using combinatorial techniques. Herein we show a different approach: First, a generative model can create diverse sets of scaffolds (or any scaffolds of interest can be used instead) and then are decorated by another generative model based on the composition of the training set. The molecules generated tend to share the same property distribution as the molecules in the training set.

As was shown in the DRD2 experiment, a decorator model trained with a few thousand molecules was able to meaningfully decorate a wide range of scaffolds (Tables 1 and 2). When compared to decoys created using ChEMBL fragments, the decorator model was able to generate molecules with a higher ratio of predicted activity. Furthermore, the decorator model was also compared to decoys built with fragments from the training set and still performed better. These results mean that the model was not only completing scaffolds with DRD2-like fragments but that it was context-aware and tried to decorate each attachment point with moieties relevant to the environment of the attachment point and also to the entire molecule. In contrast, enumerative approaches, such as fragment-based molecule generation, can generate a much larger amount of molecules, but these will not globally follow the physicochemical property distributions from the training set. We believe that these generative models can be used as an alternative to enumerative models, especially when a focused molecular generation process is required.

### Adding specific knowledge by using customized training sets

Even though most of the molecular deep generative models are trained with drug-like molecular databases such as ZINC or ChEMBL, it is known that these models can learn only to generate molecules that have specific properties (e.g., complex functional group relationships, tautomers) using specially-crafted training sets [40]. In this research, the training sets were obtained by exhaustively obtaining all possible scaffolds and decorations from all molecules. Then, in both experiments, the decorations obtained were filtered, and only those that they were fragment-like were kept. This filter, although simple, changed completely how the decorator models trained, as no examples of small scaffolds with very large decorations were in the training set. This filter contributed to making all decorations more drug-like and easily synthesizable (Table 3). Furthermore, the drug-like ChEMBL subset was additionally filtered only to allow scaffold-decorations tuples whose bonds linking the scaffolds with the decorations were following RECAP rules. More than 95% of the molecules generated, regardless of the scaffold, had all attachment point bonds compliant with the RECAP rules. This result implies that, given a scaffold, almost all molecules generated with the ChEMBL RECAP decorator can be obtained through known synthetic approaches. Moreover, the molecules generated closely matched the same physicochemical property distributions as the training set. We envision that these architectures are the first step towards having generative models able to create libraries of readily synthesizable compounds.

### Single-step vs. multi-step decorator architecture

After comparing the two architectures in two different experiments, the general trend is that, in relative terms, the multi-step architecture achieves better results than the single-step one. This trend is clear in the DRD2 experiment, where the single-step model is much less focused and generally generates a lower percent of predicted active molecules than the multi-step model (Fig. 6). Additionally, the same problem happens, although to a lesser degree, in the ChEMBL RECAP experiment (Table 3). We think that this behavior can be explained by how the training data is used by the models. For instance, when a scaffold with three attachment points is decorated by the multi-step model, it uses the information from the scaffolds in the training set with three, two, and one attachment points. Alternatively, the single-step model only uses information from similar scaffolds with three attachment points. This phenomenon can be further hinted when analyzing the attention weights [41] of both models (Additional file 2: Figure S3). In the single-step architecture, the decorator focuses on the SMILES tokens near the attachment point currently decorating. In contrast, in the multi-step architecture, there is no discernible pattern, and the model focuses on some areas of the input scaffold but unrelated to the attachment points. As it does not have to solve several problems in one run, the multi-step architecture uses information freer from the encoder than the single-step one. All of these differences make the single-step model perform slightly worse and not able to generalize as well as the multi-step model. Notwithstanding, the single-step decorator model architecture allows for a straightforward application of techniques such as reinforcement learning. We think that it may be a way to refine the model further and obtain better results.

### Comparing SMILES-based and GGNN scaffold decorators

Two approaches have been published [25, 26] that use Graph Generative Neural Networks instead of SMILES to represent molecules. They train only one model each and use a general drug-like molecular set as training data, which is preprocessed. In Li et al. Bemis-Murcko molecular frameworks [42] and in Lim et al. all possible frameworks and sub-frameworks of each molecule as given by HierS [43] are used as training data. More importantly, their scaffolds do not have explicit attachment points, and they can refine the output from the generative model by using techniques such as reinforcement learning.

The approach here described differs substantially. First and foremost, instead of using graphs, SMILES strings are used as a molecular representation. This choice offers many advantages, namely a less complex and more mature generative architecture, faster training times, and the possibility of readily using data augmentation techniques. On the other hand, the SMILES syntax requires attachment points to be explicitly defined, which adds some limitations. Second, the training set pre-processing algorithm used in this research is much more complex, and it is used as a data augmentation technique, thus being able to train models with small training sets. Third, we can create focused models (e.g., synthetic chemistry aware) without the need to use techniques such as reinforcement or transfer learning. These techniques can still be used in a later phase, if necessary. We show an alternative and fully-functional way of generating molecules from scaffolds coupled with a novel way of training models from any arbitrary molecular set.

## Conclusions

In summary, we have described a new SMILES-based molecular generative model architecture that can generate molecules from scaffolds. Alongside this, we have defined an algorithm that processes any arbitrary molecular set into a set comprised of scaffold-decorations tuples by exhaustively slicing acyclic bonds of the molecule and obtaining all possible combinations. Depending on the restrictions applied to the bonds susceptible to be sliced by the algorithm and also by filtering scaffolds that do not match certain conditions, the resulting training sets vary and allow models to be aware of medicinal or synthetic chemistry constraints. For instance, models were trained from a DRD2 modulator set (137,061 scaffold-decorations tuples obtained from a small set of 4211 molecules) and were shown to selectively decorate diverse sets of scaffolds and obtain large amounts of DRD2 predicted active molecules. Additionally, a large drug-like subset of ChEMBL was sliced only on bonds that fulfilled the RECAP rules and yielded a large 4,167,207 scaffold-decorations tuples set. Models trained with it became synthetic chemistry-aware and generated molecules that had synthetically feasible decorations and could be joined to the scaffold using known synthetic routes. We encourage other researchers to try different sets of constraints to make models aware of different properties. For instance, using reaction data to slice the training set could yield to a decorator that generates molecules with decorations joined by more complex synthetic rules. Moreover, this architecture can straightforwardly be coupled with a wide array of already reported techniques, such as temperature [44], reinforcement learning [13], and transfer learning [11] to further guide the molecule generation. In conclusion, we think that this SMILES-based generative model will become a useful addition to the already existent SMILES-based architectures and an alternative to graph-based scaffold decoration approaches.

## Methods

### Architecture details

#### Scaffold generator model

**Fig. 11:**
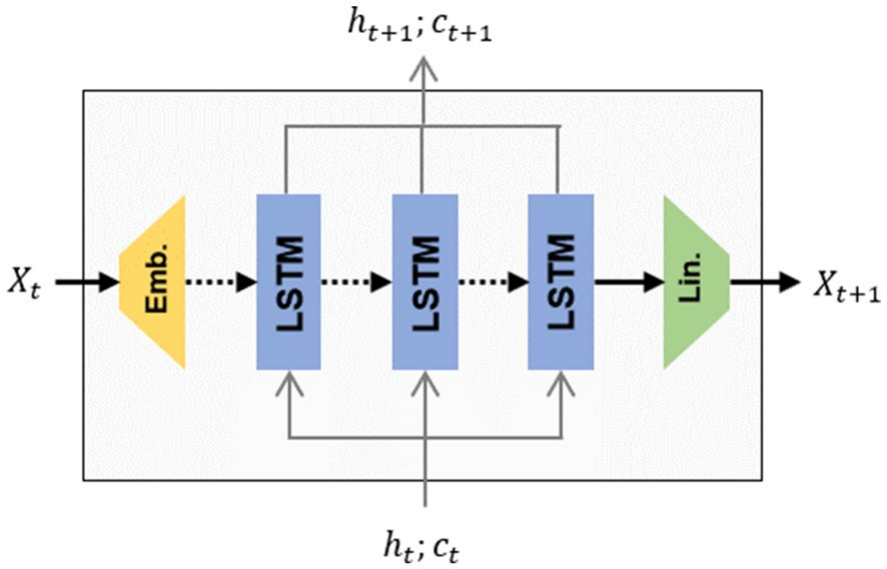
Architecture of an RNN cell used in both the scaffold generator and decorator models. Input passes through an embedding layer, three LSTM layers with 512 dimensions, and lastly, a feed-forward layer that reshapes the input to the size of the vocabulary. Dashed lines mean that a dropout layer is added during training

**Fig. 12:**
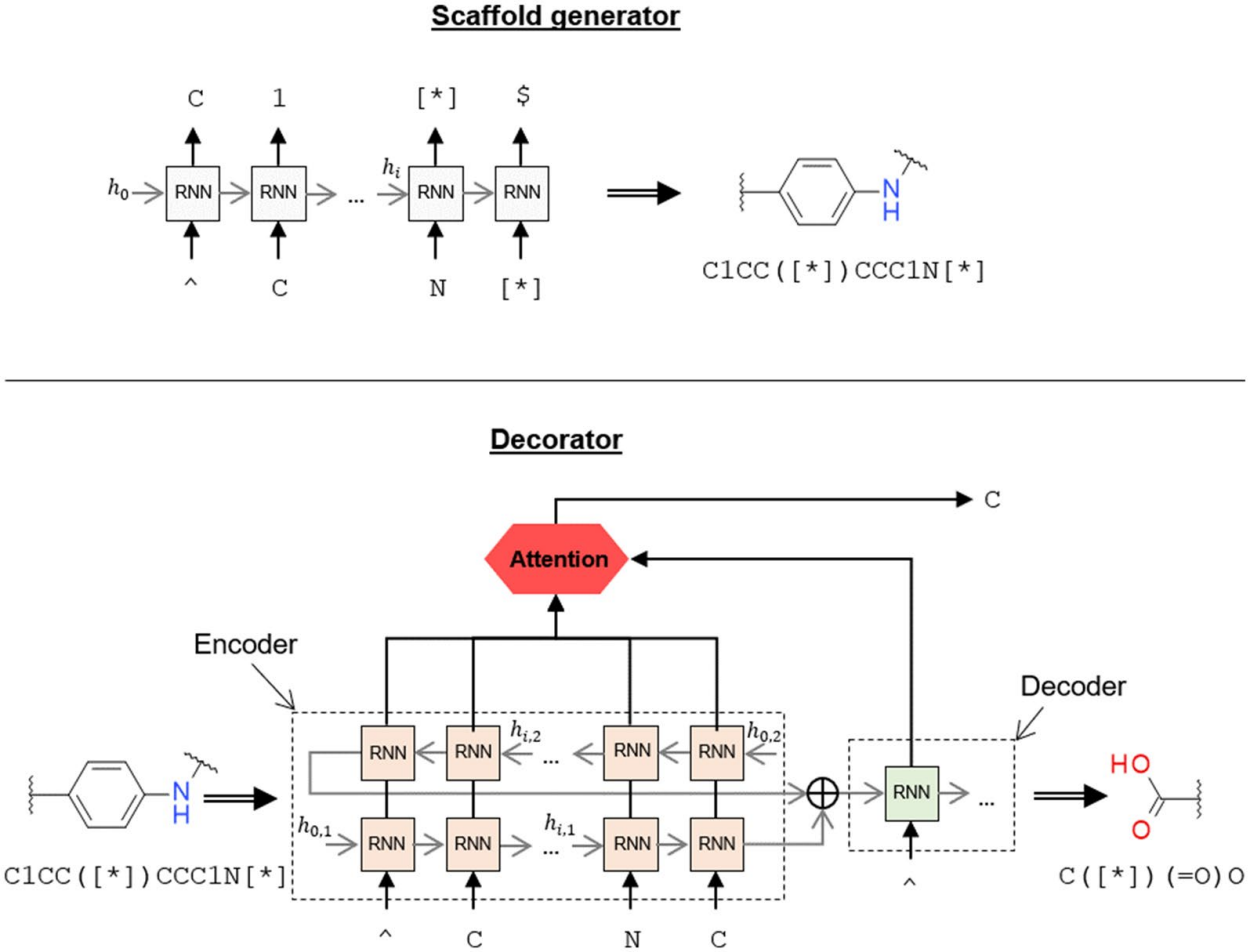
Process how the scaffold generator and the decorator sample new SMILES. The Scaffold Generator (top) samples the tokens one at a time. The decorator (bottom), on the other hand, encodes the scaffold using a bidirectional RNN. Each output state and hidden state of the encoder is summed up. The output hidden states from the encoder are input directly to the decoder. Legend: squares are instances of RNN as described in Fig. 11; gray lines hidden states in each of the sampling steps; $\oplus$ symbolizes the position-wise sum of all tensors

The scaffold generator was a randomized SMILES-based RNN similar to those already reported [10] (Figs. 11 and 12top). It featured an embedding layer, followed by three interconnected LSTM cell layers with 512 dimensions and, lastly, a linear layer that reshaped the input to the vocabulary size. Dropout layers [45] were added between all layers except for the last, which had a softmax activation function instead. The vocabulary was generated by tokenizing SMILES strings to atom tokens (e.g., “Cl”, “O”, “[nH]”), bond tokens (e.g., “=”, “#“), branching tokens (e.g., “1”, “(“) and the special attachment point token “[*]”. Batches were comprised of sequences of different lengths, so they were padded with zeroes and masked during training and sampling. A dropout = 0.2 was used between layers. The loss function for a sequence with T tokens is the Negative Log-Likelihood (NLL):

1 $$NLL\_SG_{T} := - \left[ {P\left( {X_{1} = x_{1} } \right) + \mathop \sum \limits_{i = 2}^{T} P(X_{i} = x_{i} |X_{i - 1} = x_{i - 1} , \ldots ,X_{1} = x_{1} )} \right]$$

where $X_{i}$ is a random variable with all tokens of the vocabulary as possible values at step $i$ and $x_{i}$ is the token chosen at that step. Teacher’s forcing was used to help the model learn the syntax correctly. A small hyperparameter optimization was performed using the UC-JSD, as defined in [10], to obtain the optimal values of the hyperparameters.

#### Decorator model

As previously used in NMT, the decorator model was an implementation of the simplest of the encoder-decoder architectures with attention [41, 46] (Fig. 12, bottom). The encoder was a bidirectional RNN with an embedding layer connected to three LSTM cell layers of 512 dimensions. The $c$ and $h$ hidden states for both directions were summed up together and fed to the decoder, which was a single direction RNN with three LSTM cell layers of 512 dimensions. The model also featured a global attention mechanism [41] that combined the summed outputs for both directions of the encoder in each step with the output of the current step of the decoder.

This architectural feature allowed the decoder to focus on specific regions of the input scaffold at any given decoding step. Specifically, the attention mechanism is defined as:

2 $$\begin{aligned} & AW_{i} := softmax\left( {\frac{{O_{d,i} O_{e}^{T} }}{\sqrt d }} \right) \\ & AC_{i} := AW_{i} \odot O_{e} \\ & O^{\prime}_{d,i} := tanh(W[O_{d,i} ;AC_{i} ]) \\ \end{aligned}$$

The encoded output $O^{\prime}_{d,i}$ on step $i$ was obtained by first calculating the attention weights $AW_{i}$ on that step, using the raw output of the decoder and the outputs from the encoder $O_{e}$ and performing a scaled dot product [47] and a softmax. Then, the attention context $AC_{i}$ was obtained by performing the Hadamard product (entry-wise product) between the weights and the encoder output. Lastly, $O_{d,i}$ and the attention context were concatenated and passed through a linear layer with a hyperbolic tangent activation function to convert the data back to the right shape. The last step was a linear layer to reshape the output to the vocabulary size and a softmax activation function to obtain the probabilities for each token.

The loss function was the same as the previous model with a slight difference:

3 $$NLL\_Dec_{T} := - \left[ {P\left( {X_{1} = x_{1} |S = s} \right) + \mathop \sum \limits_{i = 2}^{t} P(X_{i} = x_{i} |X_{i - 1} = x_{i - 1} , \ldots ,X_{1} = x_{1} ,S = s)} \right]$$

The NLL was dependent on the scaffold randomized SMILES used as an input. Lastly, teacher’s forcing was used on the decoder.

### Sampling process

#### Decorator model

Given that the decorator model uses randomized SMILES, the whole decoration chemical space is the union of the chemical spaces of each of the randomized SMILES of a scaffold. Consequently, the input scaffold SMILES string was randomized multiple times and sampled with each of the randomized SMILES independently. Then, the resulting half-built molecules were checked for repeats, and the process repeated until all the scaffolds were decorated. For instance, given a scaffold with 3 attachment points, if 16 randomized SMILES are generated at each stage and for each SMILES 16 decorations are sampled, the model would need to be sampled at most $\left( {16 \cdot 16} \right)^{3} = 16,777,216$ ‬ times. Due to the high number of repeats, the model always samples a much smaller number of molecules. Nevertheless, software using Apache Spark and CUDA was developed to explore the decoration chemical space of any scaffold exhaustively. It yields all the decoration combinations generated and the number of times each one has been sampled. In all the experiments, 16 randomized SMILES were generated, and each was sampled 16 times on each decoration step.

The decorator model that decorates all attachment points at once needs only one step and is much faster to sample. Consequently, 1024 randomized SMILES are generated for each scaffold, and 128 decorations are made, yielding a maximum of 131,072 possible decorations.

#### Scaffold model

For all the experiments, sampling of novel scaffolds was performed the following way: first, 10 million scaffolds were sampled from a scaffold generator model, and repeated scaffolds were filtered out. Then, scaffolds with an ECFP6 with 2048 bits Tanimoto Similarity higher than 0.7 to any molecule in the training set or that had been sampled less than 10 times (i.e., to remove outliers) were filtered out. Butina clustering [48] using the ECFP6 fingerprint with 2048 bits and a Tanimoto similarity threshold of 0.2 was performed, and $n$ scaffolds among the biggest clusters were selected for decorating.

### Result analysis

#### DRD2 model

For the DRD2 model analysis, an activity prediction model (APM) was used. This APM was trained on both the active and inactive compounds of the ExCAPE DRD2 modulator set. Stereochemistry was stripped from all compounds in the dataset, canonical SMILES were obtained, and duplicates were removed. Data were split to test, and training sets with a stratified split and the compounds were represented with ECFP6 fingerprint hashed to 2048 bits. Scikit-learn Random Forest Classifier (RF) [49] model was trained to discriminate active from inactive compounds. Optuna [50] was used for finding the optimal hyperparameters with fivefold cross-validation. The performance of the resulting model in terms of area under the curve (AUC) was 0.945.

The model was tested for improvement over decoys (i.e., randomly decorated molecules) generated from two sets of decorations. The first was comprised of 61,717 decorations from ChEMBL, obtained by slicing a small random sample of ChEMBL with the same algorithm and ensured that the decoys were drug-like and complied with the restrictions applied to the decorations in the original training set. The others were the 5532 different decorations extracted from the DRD2 training set. The random decoration process was performed one decoration at a time and ensuring that the overall molecular weight distribution was the same as that of the molecules of the training set. Lastly, two metrics were used to evaluate decorated molecules: first, the ratio of actives in a set of decorations (either generated or decoys) of the same scaffold given $p_{active} \ge 0.5$; then, the Enrichment Over Random, calculated as the $ratio_{actives} /ratio_{decoys}$.

#### ChEMBL model

The ChEMBL models were tested whether they were able to add decorations whose bond uniting them to the scaffold followed the RECAP rules. This condition was assessed by implementing the RECAP rules using the SMARTS syntax and checking each molecule, whether the bond joining the attachment point and the decoration complied with the RECAP rules. Additionally, the decorations generated were checked where they were in the In-Stock Fragment subset of ZINC by comparing the canonical SMILES.

### Technical details

Python 3.6.9 was used to develop all software. Mainly, PyTorch 1.4 [51] was used to develop all generative models; RDKit [52] version 2019.03.3.0 was used to work with molecules, calculate fingerprints and perform Butina clustering; Apache Spark [53] 2.4 was used to create and manage all the data. All models were trained with Nvidia Tesla V-100 cards using CUDA 10. The TMAPs were generated with version 1.0 of the library [34].

## Supplementary information

**Additional file 1.** Additional Methods.

**Additional file 2.** Additional Tables and Figures.

## Data Availability

The code used to train and benchmark both the scaffold^1^ and decorator generative models is publicly released under an MIT License.^2^ The TMAPs and all molecular datasets are available on the Reymond group website^3^ and Zenodo, respectively.^4^

## Abbreviations

ADAM: Adaptative moment estimation
APM: Activity prediction model
DRD2: Dopamine receptor D2
ECFP: Enhanced connectivity fingerprint
GAN: Generative Adversarial Network
GGNN: Graph generative neural network
HBA: Hydrogen bond acceptor
HBD: Hydrogen bond donor
JSD: Jensen-Shannon divergence
LSTM: Long short-term memory
MMP: Matched molecular pair
NLL: Negative log-likelihood
NMT: Neural machine translation
QED: Quantitative estimate of drug-likeness
RECAP: Retrosynthetic combinatorial analysis procedure
RNN: Recurrent neural network
SMARTS: SMILES arbitrary target specification
SMILES: Simple molecular input line entry system
SA Score: Synthetic Accessibility Score
SVM: Support vector machine
TMAP: Tree Map
UC-JSD: Uniformity-completeness JSD
VAE: Variational autoencoder
