# Supplementary material for: SMILES-based deep generative scaffold decorator for de-novo drug design
Source: J Cheminform. 2020 May 29;12:38. doi: 10.1186/s13321-020-00441-8 (PMC7260788; doi:10.1186/s13321-020-00441-8)
Supplement: Supplementary file 1 — Additional file 1. Additional Methods. [file 13321_2020_441_MOESM1_ESM.pdf]

Additional methods

SMILES-based deep generative scaffold decorator for de-novo drug design

Josep Arús-Pous[§⊥*], Atanas Patronov[§], Esben Jannik Bjerrum[§], Christian Tyrchan[‖], Jean-Louis Reymond[⊥], Hongming Chen[¥], Ola Engkvist[§].

§ Molecular AI, Hit Discovery, Discovery Sciences, BioPharmaceutical R&D, AstraZeneca, Gothenburg, Sweden.

‖ Medicinal Chemistry, Respiratory Inflammation, and Autoimmune (RIA), BioPharmaceutical R&D, AstraZeneca, Gothenburg, Sweden.

⊥ Department of Chemistry and Biochemistry, University of Bern, Freiestrasse 3, 3012 Bern, Switzerland.

¥ Chemistry and Chemical Biology Centre, Guangzhou Regenerative Medicine and Health - Guangdong Laboratory, Guangzhou, China

* Corresponding author: josep.arus@dcb.unibe.ch

S1. Databases

DRD2 modulators

A set of 4,613 human DRD2 active modulators ($pXC50 \geq 5$) obtained from ExCAPE DB [1] was downloaded from the official website and was cleaned using a process very similar to [2], which had the following steps: First, the MolVS 0.1.1 library [3] was used to sanitize all molecules, remove duplicates, stereochemistry, salts and all fragments except for the largest were removed. Then, all molecules containing heavy atom types other than (C, N, O, S, Cl, Br, F) were removed. After, a series of filters were applied sequentially to remove outliers (**Table 1**). The ranges were determined by a cutoff of 0.5% in both sides of the histograms of each property.

Filter	Range allowed	Size after
**Full DRD2 set**	-	4,613
**Standardization**	MolVS complete sanitization and filtered molecules with heavy atoms other than C, N, O S, Cl, Br and F.	4,315
**Ring count (SSSR)**	$1 \leq \text{RC} \leq 8$	4,304
**# tokens**	$\text{NT} \leq 70$	4,219
**Token filter**	Removed SMILES with non-ring tokens with less than 0.5 % abundance in the dataset.	4,211

Table 1: Filters applied to DRD2 modulator set from ExCAPE DB in order (from top to bottom). The first entry is not a filter but represents the initial state.

ChEMBL subset

The ChEMBL 25 database [4] was obtained from the official website, and the same process as in the DRD2 set was used to filter the database but with different cutoffs and some additional descriptors (**Table 2**). Specifically, all molecules bigger than 40 heavy atoms and with less than two rings or with ring size different than 5 or 6 were discarded. Also, a restrictive QED [5] filter was applied that removed around 350.000 compounds. Lastly, molecules whose

SMILES was too complicated or that included tokens that seldom appeared in the dataset were filtered. This process ensured a database with fewer outliers.

Filter	Range allowed	Size after
**Full ChEMBL 25**	-	1,870,461
**Standardization**	MolVS complete sanitization and filtered molecules with heavy atoms other than C, N, O S, Cl, Br, and F.	1,647,004
**Heavy atom count (HAC)**	$15 \leq \text{HAC} \leq 40$	1,460,210
**Ring count (SSSR)**	$2 \leq \text{RC} \leq 5$	1,348,432
**Size of largest ring**	$5 \leq \text{SLR} \leq 6$	1,284,673
**Max aliphatic C chain size**	$ACC \leq 3$	1,241,337
**# C atoms / HAC**	ratio ≥ 0.6	1,198,308
**QED [5]**	$QED > 0.5$	845,679
**# tokens**	$\text{NT} \leq 60$ (used 0.1% cut-off)	831,450
**# tokens / HAC**	ratio ≤ 2.0	830,085
**Token filter**	All non-ring tokens with less than 0.05% canonical SMILES strings were removed.	827,098

Table 2: Filters applied to ChEMBL 25 database in order (from top to bottom). The first entry represents the ChEMBL 25 initial state. The cut-offs were set arbitrarily, focusing only on keeping highly drug-like compounds.

ZINC fragments

The In-Stock Fragment subset of ZINC was obtained from the official website, and was further processed with RDKit to remove stereochemistry, obtain canonical SMILES, and remove repeated molecules. The final size of the subset was 541,281 molecules. This database was used only to check whether decorations from the ChEMBL model were readily purchasable. As the database holds molecules with more than 3 heavy atoms, any smaller decoration was considered to be automatically in the database.

S2. Training details

DRD2 models

The decorator models (multi-step and single-step) were trained with a split training-validation set of (131,241; 5,820) scaffold-decorations. The model was trained for 100 epochs, a batch size of 64, with exponential learning rate decay with a starting value of 10^{-3} down to 10^{-5}. The ADAM optimizer with parameters $\beta_1 = 0.9,\ \beta_2 = 0.999, \epsilon = \ 10^{-8}$ was used throughout. The models took roughly 1 day to train each.

The scaffold generator model was trained on a subset of the scaffolds in the training set, which included all scaffolds with at least two attachment points and that the shortest path between all attachment points passed through a ring atom. This set amounted to 9,925 scaffolds, which were divided into a training-validation sets of (9,425; 500) scaffolds each. The model was trained for 500 epochs with the same exponential learning rate and optimizer specified before, but with a batch size of 8. The model took roughly 3 hours to train, and the best epoch was chosen using the UC-JSD, as specified in [2].

ChEMBL models

The decorator models were trained using the same hyperparameters as the decorator models in the previous section, except for the batch size, which was increased to 512. The training-validation set split was (4,119,080; 48,127). As the training set was very large, models took 50 minutes to train each epoch, amounting to a total of 3-4 days each.

The scaffold generator was trained with the same hyperparameters as the previous one but with a batch size of 32. The training set was obtained the same way as the other decorator model, yielding a total of 167,099 scaffolds, which was then split to (162,099; 5000) for the training and validation sets, respectively. The model took 2 days and 10 hours to train, and the best epoch was chosen using the UC-JSD as before.

A note on training duration

The models in this research were trained using randomized SMILES. As shown in [2], these models give good results when just trained a few epochs, but they can be trained for very long periods and obtain models that make fewer mistakes. Other published approaches [7], [8] use graph generative models, which are substantially slower than SMILES models, but in their applications, only train their models for a tiny amount of steps. Thus the total training time is lower. For instance, the decorator model from [7] was trained for 50,000 steps and took 20 hours, whereas our ChEMBL decorator model was trained for $4167207 \cdot \frac{100}{512} = 804{,}508$ steps during 3-4 days. An equivalent number of steps for the GGNN model would have taken the model $\frac{20}{50000} \cdot \frac{804507}{24} \approx 13.4$ days, assuming that the GPUs used were equivalent.

References

[1] J. Sun *et al.*, "ExCAPE-DB: an integrated large scale dataset facilitating Big Data analysis in chemogenomics," *Journal of Cheminformatics*, vol. 9, no. 1, p. 17, Mar. 2017, doi: 10.1186/s13321-017-0203-5.

[2] J. Arús-Pous *et al.*, "Randomized SMILES strings improve the quality of molecular generative models," *Journal of Cheminformatics*, vol. 11, no. 1, p. 71, Nov. 2019, doi: 10.1186/s13321-019-0393-0.

[3] M. Swain and J. Meyers, *mcs07/MolVS: MolVS v0.1.1*. Zenodo, 2018.

[4] A. Gaulton *et al.*, "The ChEMBL database in 2017," *Nucleic Acids Res*, vol. 45, no. Database issue, pp. D945–D954, Jan. 2017, doi: 10.1093/nar/gkw1074.

[5] G. R. Bickerton, G. V. Paolini, J. Besnard, S. Muresan, and A. L. Hopkins, "Quantifying the chemical beauty of drugs," *Nature Chemistry*, vol. 4, no. 2, pp. 90–98, Feb. 2012, doi: 10.1038/nchem.1243.

[6] D. P. Kingma and J. Ba, "Adam: A Method for Stochastic Optimization," *arXiv:1412.6980 [cs]*, Jan. 2017, Accessed: Feb. 18, 2020. [Online]. Available: http://arxiv.org/abs/1412.6980.

[7] Y. Li, J. Hu, Y. Wang, J. Zhou, L. Zhang, and Z. Liu, "DeepScaffold: A Comprehensive Tool for Scaffold-Based De Novo Drug Discovery Using Deep Learning," *J. Chem. Inf. Model.*, vol. 60, no. 1, pp. 77–91, Jan. 2020, doi: 10.1021/acs.jcim.9b00727.

[8] J. Lim, S.-Y. Hwang, S. Moon, S. Kim, and W. Y. Kim, "Scaffold-based molecular design with a graph generative model," *Chem. Sci.*, vol. 11, no. 4, pp. 1153–1164, Jan. 2020, doi: 10.1039/C9SC04503A.
