# Supplementary material for: SMILES-based deep generative scaffold decorator for de-novo drug design
Source: J Cheminform. 2020 May 29;12:38. doi: 10.1186/s13321-020-00441-8 (PMC7260788; doi:10.1186/s13321-020-00441-8)
Supplement: Supplementary file 2 — Additional file 2. Additional Tables and Figures. [file 13321_2020_441_MOESM2_ESM.pdf]

# Additional figures and tables

## SMILES-based deep generative scaffold decorator for de-novo drug design

Josep Arús-Pous[§⊥*], Atanas Patronov[§], Esben Jannik Bjerrum[§], Christian Tyrchan[‖], Jean-Louis Reymond[⊥], Hongming Chen[¥], Ola Engkvist[§].

§ Molecular AI, Hit Discovery, Discovery Sciences, BioPharmaceutical R&D, AstraZeneca, Gothenburg, Sweden.

‖ Medicinal Chemistry, Respiratory Inflammation, and Autoimmune (RIA), BioPharmaceutical R&D, AstraZeneca, Gothenburg, Sweden.

⊥ Department of Chemistry and Biochemistry, University of Bern, Freiestrasse 3, 3012 Bern, Switzerland.

¥ Chemistry and Chemical Biology Centre, Guangzhou Regenerative Medicine and Health - Guangdong Laboratory, Guangzhou, China

* Corresponding author: josep.arus@dcb.unibe.ch

# Additional Tables

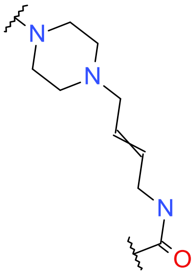

1

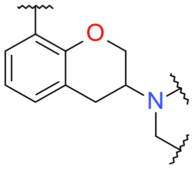

2

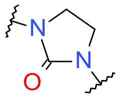

3

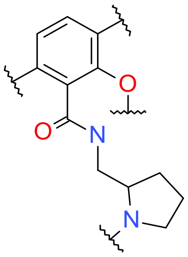

4

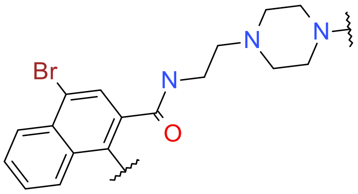

5

| | Generated | | ChEMBL decoys | | | DRD2 decoys | | |
|---|---|---|---|---|---|---|---|---|
| S | *Total* | *% act.* | *% act.* | *% diff* | *EOR* | *% act.* | *% diff* | *EOR* |
| 1 | 11,628 | 47.2 % | 0.9 % | 46.3 % | 52.6 | 4.0 % | 43.2 % | 11.7 |
| 2 | 10,483 | 29.9 % | 8.0 % | 21.9 % | 3.7 | 19.5 % | 10.4 % | 1.5 |
| 3 | 77,548 | 26.1 % | 0.8 % | 25.3 % | 32.2 | 15.6 % | 10.5 % | 1.7 |
| 4 | 844 | 25.2 % | 0.4 % | 24.9 % | 69.3 | 1.0 % | 24.2 % | 25.1 |
| 5 | 182 | 28.0 % | 9.9 % | 18.1 % | 2.8 | 15.1 % | 12.9 % | 1.9 |

**Table S1:** Scaffolds obtained from the validation set of the DRD2 dataset alongside the results from the decoration process using the single-step decoration model. **Legend:** Total number of molecules sampled (Total); Percent of generated molecules that are predicted as active ($p_{active} \geq 0.5$) by the APM (% act); For both the decoys decorated with ChEMBL fragments and DRD2 fragments from the training set: Percent of predicted active decoys ($p_{active} \geq 0.5$) (% act); Difference between the generated predicted active percent and the predicted active percent of the decoys ( % Diff); Enrichment Over Random ($percent_{active}/percent_{decoy}$) (EOR).

| | Generated | | ChEMBL decoys | | | DRD2 decoys | | |
|---|---|---|---|---|---|---|---|---|
| S | *Total* | *Sampled* | *Total* | *Overlap* | *% overlap* | *Total* | *Overlap* | *% overlap* |
| 1 | 1,728 | 26,759 | 25,724 | 1 | 0.1 % | 24,793 | 3 | 0.2 % |
| 2 | 1,626 | 61,115 | 59,084 | 8 | 0.5 % | 58,029 | 19 | 1.1 % |
| 3 | 10,666 | 38,074 | 36,554 | 0 | 0 % | 34,353 | 0 | 0 % |
| 4 | 262 | 50,163 | 46,531 | 0 | 0 % | 45,892 | 2 | 0.8 % |
| 5 | 18 | 1,791 | 841 | 0 | 0 % | 810 | 7 | 38.9 % |

**Table S2**: Additional sampling statistics for the multi-step model sample of the five validation set scaffolds. **Legend:** Total number of unique molecules obtained with each sampling approach (Total); Number of sampled molecules in total using the decorator model (Sampled); Nomber of molecules that overlap between a given decoy set and the molecules generated with a decorator (Overlap); Ratio between the overlap and the total obtained (% overlap).

| | Generated | | ChEMBL decoys | | | DRD2 decoys | | |
|---|---|---|---|---|---|---|---|---|
| S | *Total* | *Sampled* | *Total* | *Overlap* | *% overlap* | *Total* | *Overlap* | *% overlap* |
| 1 | 11,628 | 124,225 | 118,331 | 14 | 0.1 % | 109,585 | 21 | 0.2 % |
| 2 | 10,483 | 129,515 | 126,403 | 3 | 0.0 % | 124,635 | 39 | 0.4 % |
| 3 | 77,548 | 117,598 | 114,277 | 19 | 0.0 % | 104,535 | 44 | 0.1 % |
| 4 | 844 | 130,914 | 120,527 | 1 | 0.1 % | 117,799 | 77 | 9.1 % |
| 5 | 182 | 130,988 | 38,040 | 4 | 2.2 % | 27,823 | 58 | 31.9 % |

**Table S3**: Additional sampling statistics for the single-step model sample of the five validation set scaffolds. **Legend:** Total number of unique molecules obtained with each sampling approach (Total); Number of sampled molecules in total using the decorator model (Sampled); Nomber of molecules that overlap between a given decoy set and the molecules generated with a decorator (Overlap); Ratio between the overlap and the total obtained (% overlap).

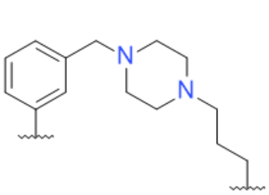

**6**

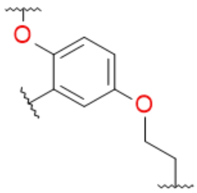

**7**

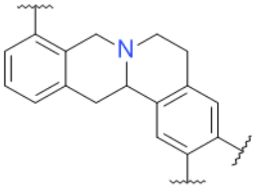

**8**

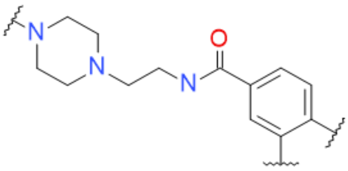

**9**

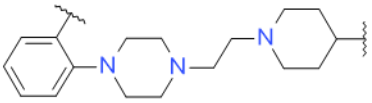

**10**

| | Generated | | ChEMBL decoys | | | DRD2 decoys | | |
|---|---|---|---|---|---|---|---|---|
| S | *Total* | *% act.* | *% act.* | *% diff* | *EOR* | *% act.* | *% diff* | *EOR* |
| **6** | 15,885 | 78.1 % | 48.3 % | 29.7 % | 1.6 | 64.6 % | 13.5 % | 1.2 |
| **7** | 49,799 | 18.2 % | 1.0 % | 17.2 % | 18.1 | 10.7 % | 7.5 % | 1.7 |
| **8** | 2,194 | 90.2 % | 44.1 % | 46.1 % | 2.0 | 48.4 % | 41.8 % | 1.9 |
| **9** | 1,525 | 8.8 % | 2.9 % | 5.8 % | 3.0 | 6.8 % | 1.9 % | 1.3 |
| **10** | 9,278 | 96.4 % | 89.5 % | 6.9 % | 1.1 | 92.6 % | 3.8 % | 1.0 |

**Table S4:** Non-dataset scaffolds obtained from a generative model (see methods) and results of the decoration of each of them using the single-step decoration model. **Legend:** Total number of molecules sampled (Total); Percent of generated molecules that are predicted as active ($p_{active} \geq 0.5$) by the APM (% act); For both the decoys decorated with ChEMBL fragments and DRD2 fragments from the training set: Percent of predicted active decoys ($p_{active} \geq 0.5$) (% act); Difference between the generated predicted active percent and the predicted active percent of the decoys (% Diff); Enrichment Over Random ($percent_{active}/percent_{decoy}$) (EOR).

| | Generated | | ChEMBL decoys | | | DRD2 decoys | | |
|---|---|---|---|---|---|---|---|---|
| S | *Total* | *Sampled* | *Total* | *Overlap* | *% overlap* | *Total* | *Overlap* | *% overlap* |
| **6** | 1,864 | 21,947 | 20,665 | 8 | 0.4 % | 19,857 | 26 | 1.4 % |
| **7** | 15,724 | 528,094 | 501,054 | 15 | 0.1 % | 480,975 | 100 | 0.6 % |
| **8** | 2,178 | 70,617 | 62,618 | 105 | 4.8 % | 60,091 | 175 | 8.0 % |
| **9** | 5,362 | 143,101 | 124,532 | 32 | 0.6 % | 117,511 | 70 | 1.3 % |
| **10** | 1,012 | 13,491 | 10,843 | 7 | 0.7 % | 10,058 | 40 | 3.9 % |

**Table S5**: Additional sampling statistics for the multi-step model sample of the five non-dataset scaffolds. **Legend:** Total number of unique molecules obtained with each sampling approach (Total); Number of sampled molecules in total using the decorator model (Sampled); Nomber of molecules that overlap between a given decoy set and the molecules generated with a decorator (Overlap); Ratio between the overlap and the total obtained (% overlap).

| | Generated | | ChEMBL decoys | | | DRD2 decoys | | |
|---|---|---|---|---|---|---|---|---|
| S | *Total* | *Sampled* | *Total* | *Overlap* | *% overlap* | *Total* | *Overlap* | *% overlap* |
| **6** | 15,885 | 127,281 | 113,932 | 215 | 1.4 % | 100,916 | 922 | 5.8 % |
| **7** | 49,799 | 126,284 | 124,656 | 17 | 0.0 % | 123,476 | 79 | 0.1 % |
| **8** | 2,194 | 130,970 | 114,531 | 133 | 6.1 % | 108,146 | 220 | 10.0 % |
| **9** | 1,525 | 130,862 | 114,634 | 6 | 0.4 % | 108,974 | 31 | 2.0 % |
| **10** | 9,278 | 129,861 | 91,142 | 177 | 1.9 % | 73,660 | 1033 | 11.1 % |

**Table S6**: Additional sampling statistics for the single-step model sample of the five non-dataset scaffolds. **Legend:** Total number of unique molecules obtained with each sampling approach (Total); Number of sampled molecules in total using the decorator model (Sampled); Nomber of molecules that overlap between a given decoy set and the molecules generated with a decorator (Overlap); Ratio between the overlap and the total obtained (% overlap).

# Additional figures

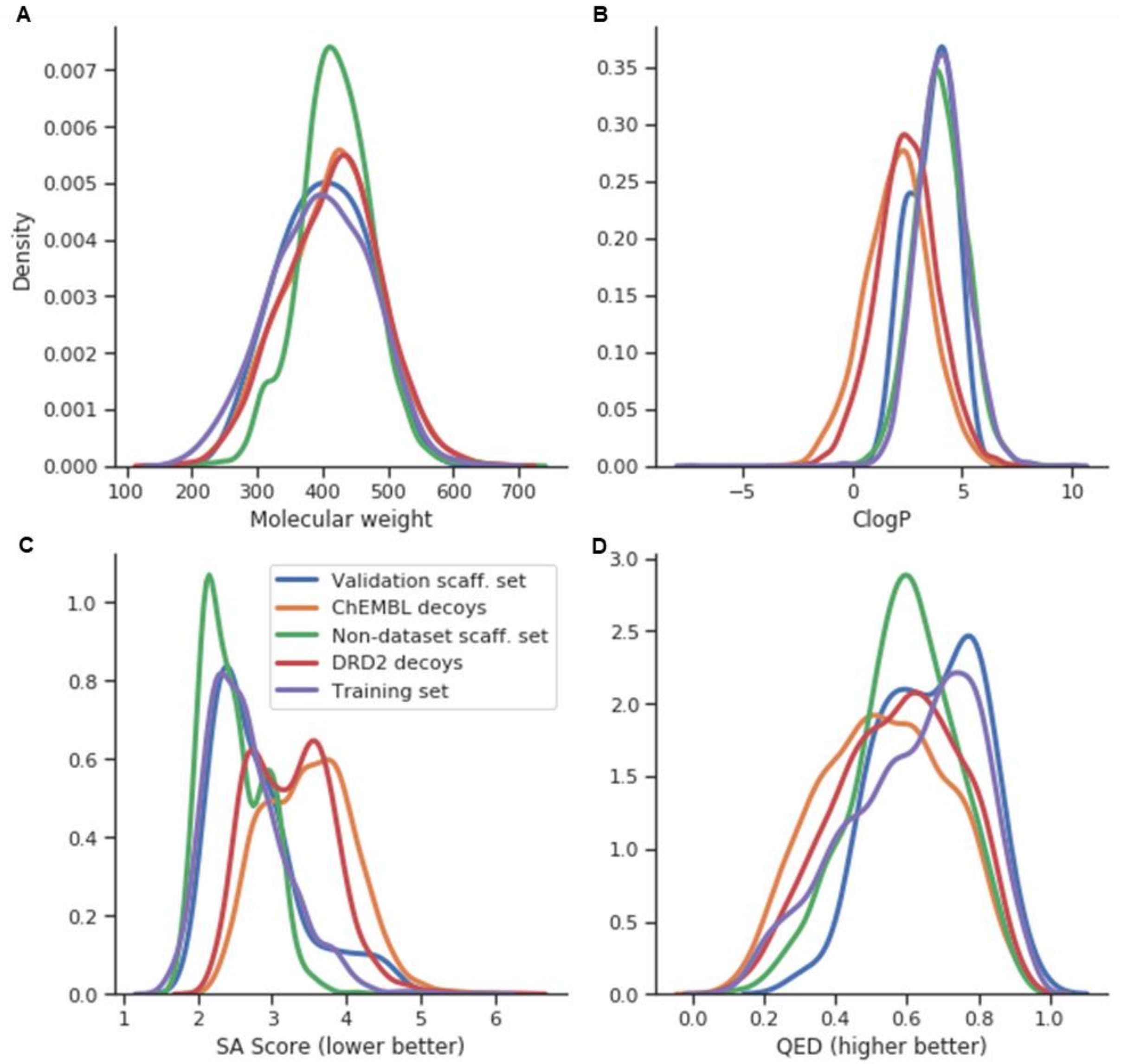


**Figure S1:** Histograms of different descriptors calculated in three sets of molecules obtained from the DRD2 multi-step decorator model: Generated molecules from validation set scaffolds (blue), generated molecules from non-dataset scaffolds (green), training set molecules (purple) and the two decoy sets (ChEMBL – orange, DRD2 – red). A) Molecular weight (Da); B) ClogP; C) Synthetic Accessibility Score; D) Quantitative Estimate of Drug Likeness (QED).

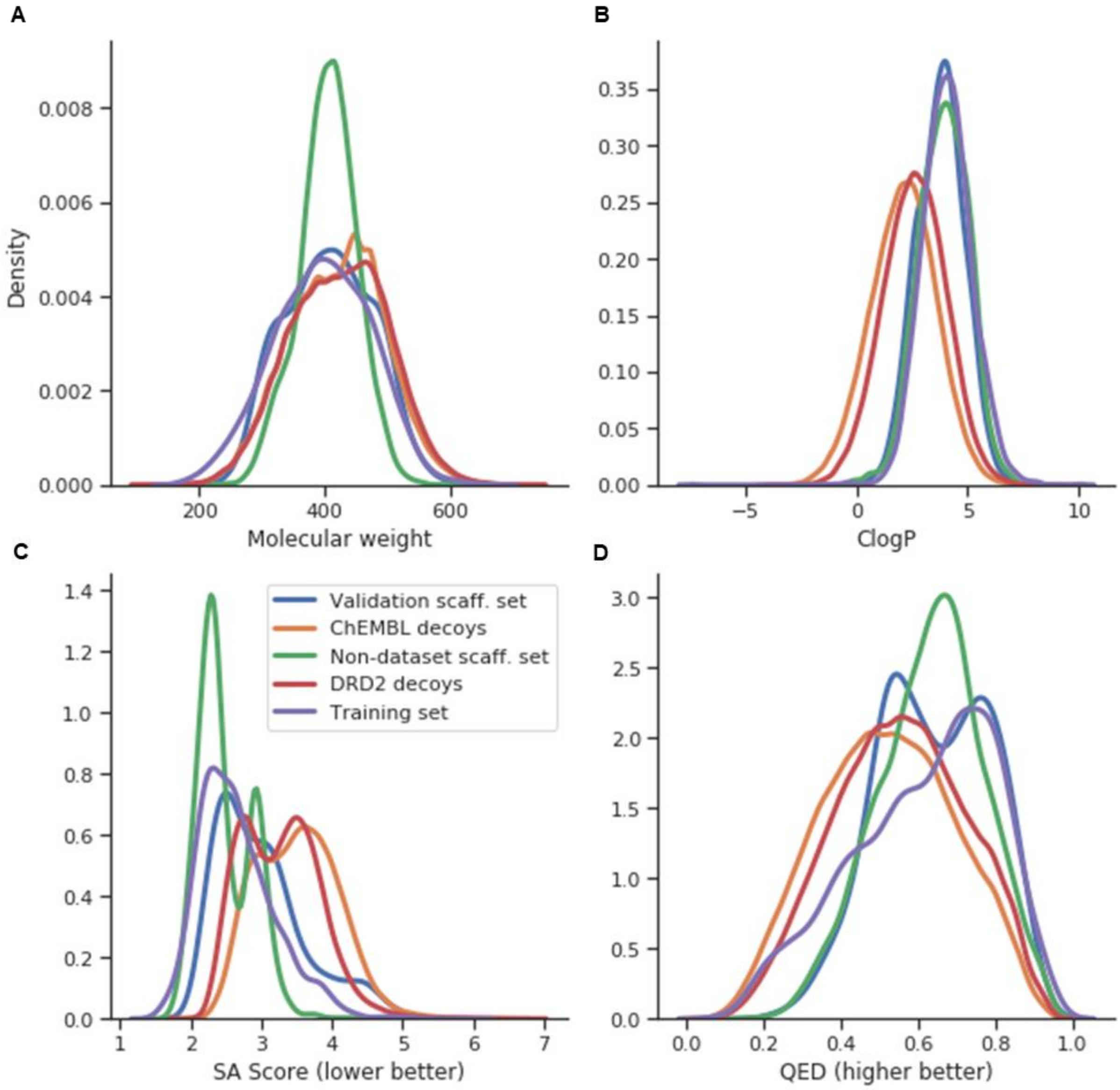


**Figure S2:** Histograms of different descriptors calculated in three sets of molecules obtained from the DRD2 single-step decorator model: Generated molecules from validation set scaffolds (blue), generated molecules from non-dataset scaffolds (green), training set molecules (purple) and the two decoy sets (ChEMBL – orange, DRD2 – red). A) Molecular weight (Da); B) ClogP; C) Synthetic Accessibility Score; D) Quantitative Estimate of Drug Likeness (QED).

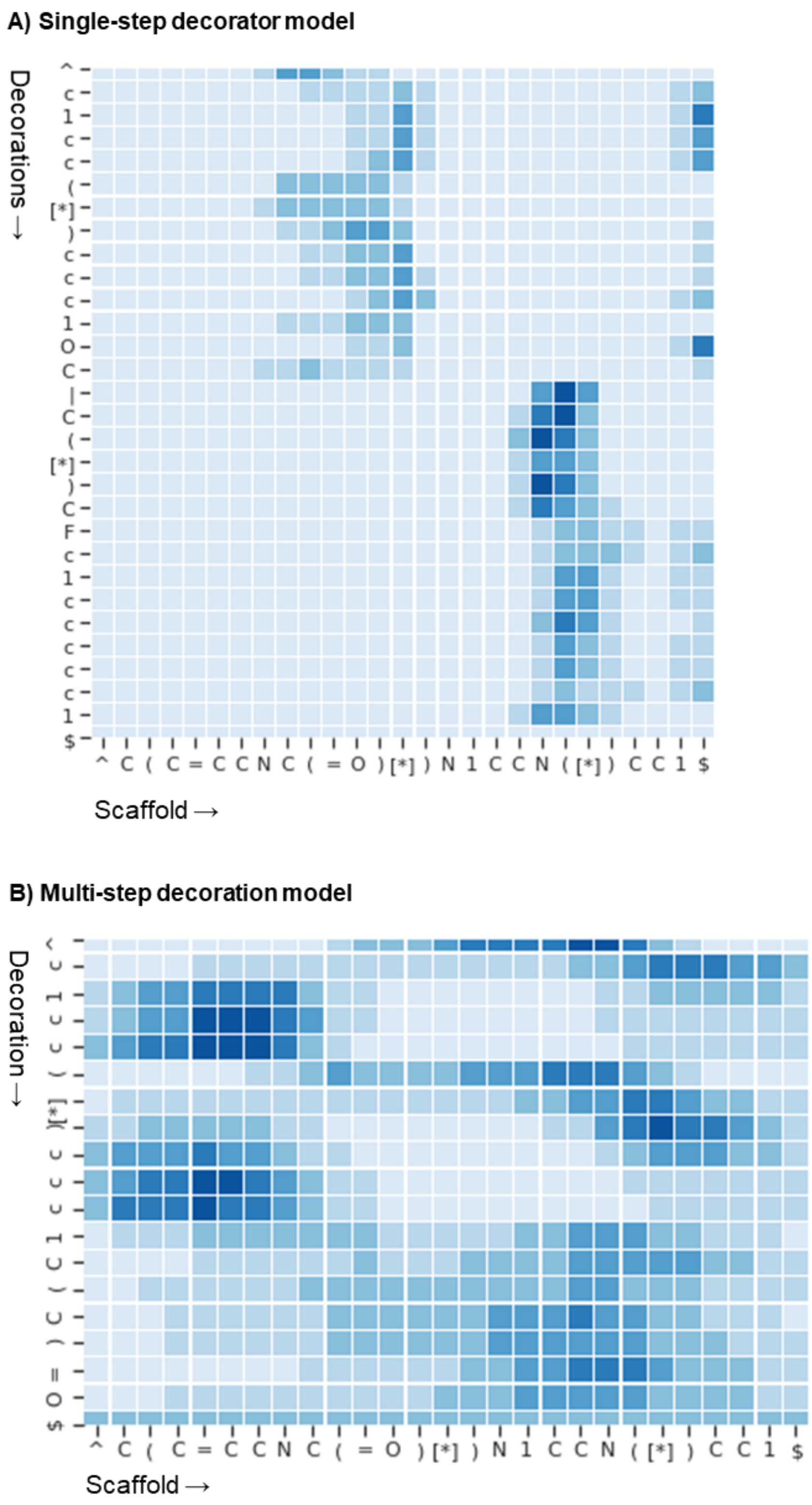


**Figure S3:** Attention weight heat maps for the same randomized SMILES of the scaffold (1) for the single-step (A) and multi-step (B) decoration DRD2 models. Notice how in the single-step model, attention weights usually are focused around the attachment point of the decoration being generated. On the other hand, the multi-step model attention weights have no human-discernible pattern.

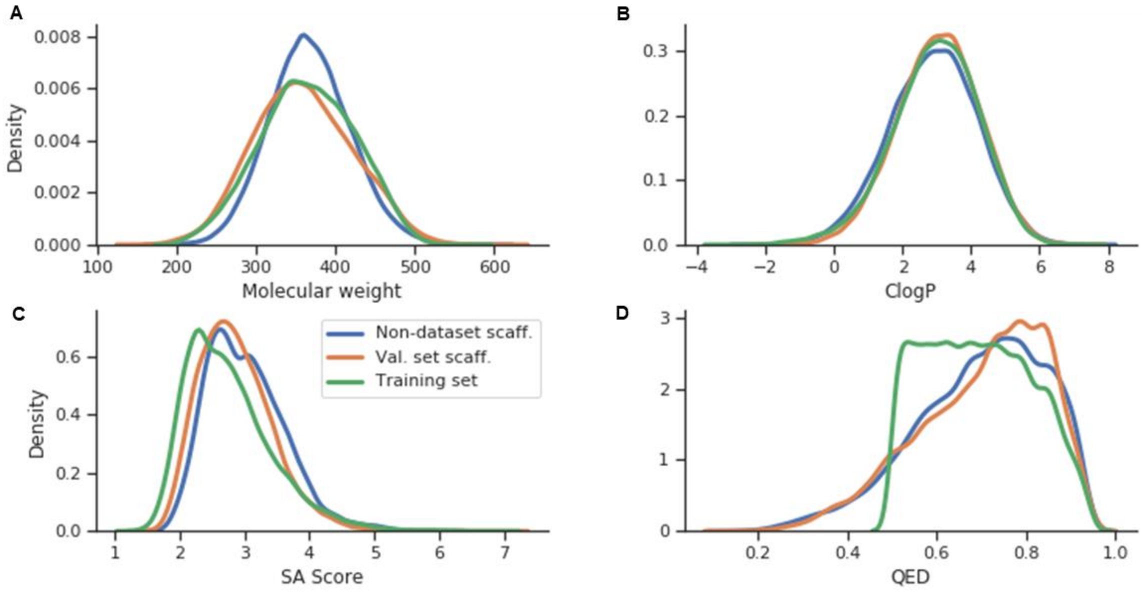


**Figure S4:** Histograms of different descriptors calculated in three sets of molecules obtained from the ChEMBL single-step decorator model: Generated molecules from non-dataset scaffolds (blue), generated molecules from validation set scaffolds (orange) and training set molecules (green). A) Molecular weight (Da); B) ClogP; C) Synthetic Accessibility Score; D) Quantitative Estimate of Drug Likeness (QED). Notice that one of the filtering conditions of the ChEMBL subset was that the molecules had $QED > 0.5$.
